# Selective Isolation of *Eggerthella lenta* from Human Faeces and Characterisation of the Species Prophage Diversity

**DOI:** 10.3390/microorganisms10010195

**Published:** 2022-01-17

**Authors:** Colin Buttimer, Francesca Bottacini, Andrey N. Shkoporov, Lorraine A. Draper, Paul Ross, Colin Hill

**Affiliations:** 1 APC Microbiome Ireland, University College Cork, T12 YT20 Cork, Ireland; colin.buttimer@ucc.ie (C.B.); andrey.shkoporov@ucc.ie (A.N.S.); L.Draper@ucc.ie (L.A.D.); P.ross@ucc.ie (P.R.); 2 Department of Biological Sciences, Munster Technological University, T12 P928 Cork, Ireland; francesca.bottacini@mtu.ie; 3 School of Microbiology, University College Cork, T12 YN60 Cork, Ireland

**Keywords:** Actinobacteria, *Eggerthella* *lenta*, bacterial isolation, prophage diversity, diversity generating retroelements

## Abstract

*Eggerthella lenta* is an anaerobic, high GC, Gram-positive bacillus commonly found in the human digestive tract that belongs to the class Coriobacteriia of the phylum Actinobacteria. This species has been of increasing interest as an important player in the metabolism of xenobiotics and dietary compounds. However, little is known regarding its susceptibility to bacteriophage predation and how this may influence its fitness. Here, we report the isolation of seven novel *E. lenta* strains using cefotaxime and ceftriaxone as selective agents. We conducted comparative and pangenome analyses of these strains and those publicly available to investigate the diversity of prophages associated with this species. Prophage gene products represent a minimum of 5.8% of the *E. lenta* pangenome, comprising at least ten distantly related prophage clades that display limited homology to currently known bacteriophages. All clades possess genes implicated in virion structure, lysis, lysogeny and, to a limited extent, DNA replication. Some prophages utilise tyrosine recombinases and diversity generating retroelements to generate phase variation among targeted genes. The prophages have differing levels of sensitivity to the CRISPR/cas systems of their hosts, with spacers from 44 *E. lenta* isolates found to target only five out of the ten identified prophage clades. Furthermore, using a PCR-based approach targeting the prophage *attP* site, we were able to determine that several of these elements can excise from the host chromosome, thus supporting the notion that these are active prophages. The findings of this study provide further insights into the diversity of prophages infecting species of the phylum Actinobacteria.

## 1. Introduction

The human gastrointestinal tract hosts a wide variety of microorganisms (bacteria, archaea, yeasts, protists and viruses) capable of extending the metabolic capabilities of the human body, with bacterial numbers in the human gut estimated to be at 10^11^ CFU/g of faeces [1,2]. These organisms are capable of extending the metabolic potential of the human superorganism, playing an essential role in the digestion of polysaccharides, the synthesis of vitamins and amino acids, as well as the modification of endogenous compounds [3]. Additionally, they play an important role in the metabolism of xenobiotics (including drugs, dietary compounds and environmental toxins), impacting the bioavailability, activity and toxicity of these compounds [4,5]. More than fifty different pharmaceutical compounds have been identified as susceptible to such metabolic alterations [6]. One human gut commensal bacterium implicated in these bioconversion processes is *Eggerthella lenta* (previously known as *Eubacterium lentum*) [7]. This is a Gram-positive, high GC, anaerobic, non-spore former bacterium belonging to the phylum Actinobacteria of the family Eggerthellaceae (formerly placed in the family *Coriobacteriaceae*) of the class Coriobacteriia [8].

A well-described example of drug metabolism by a gut commensal bacterium is the ability of *E. lenta* to reduce the medicinally important cardenolide digoxin to an inactive derivative dihydrodigoxin [5,9,10,11,12]. This cardenolide is commonly used to treat heart failure and arrhythmia. However, more than 10% of individuals excrete a significant amount of dihydrodigoxin when given digoxin [13]. This intra-individual variation in the conversion and inactivation of this drug is likely affected by the levels of resident *E. lenta* in the gut and whether strains harbour a specific genetic cluster (composed of *cgr1* and *cgr2*) encoding for the necessary metabolic capabilities implicated in digoxin inactivation. A qPCR-based assay utilising a particular marker gene (*elnmrk1*) for the presence of *E. lenta* indicated its presence in the stool of 81.6% of tested individuals (*n* = 158), corresponding to anywhere between 1 × 10^3^ to 1 × 10^8^ genome copies/gram. In comparison, examination of these same individuals by qPCR targeting the *cgr2* gene of *E. lenta* showed its prevalence among 74.7% of tested people [11].

Certain *E. lenta* strains can also inactivate the drug L-dopa, an amino acid precursor of dopamine used in the treatment of Parkinson’s disease, as the bacterium is capable of dehydroxylating the L-dopa metabolite dopamine to m-tyramine [14]. Additionally, this bacterium also reduces the uptake of dietary phytochemicals by potentially impeding resveratrol uptake by conversion to dihydroresveratrol [15]. However, not all gut metabolism due to *E. lenta* is detrimental to the host, as the bacterium has been shown to also convert plant-derived lignans to the bioactive enterolignans as part of a four species bacterial consortium [16]. It is also worth noting that the impact of *E. lenta* on human health goes beyond dietary and xenobiotic gut metabolism, as this bacterium has been occasionally identified as a pathobiont associated with bacteraemia as well as responsible for organ and soft tissue infections [17,18,19].

Bacteriophages (phages) are viruses that specifically infect bacteria, with their numbers estimated to be equal or less than their bacterial hosts in the human gut. They are also understood to influence the composition and function of the gut microbiome due to phage predation [1,20], and alterations in prophage activation resulting in lytic phage infections have been correlated with changes in the microbiome composition, that in turn can affect host health [21].

Phages predominantly infect their hosts following two life cycles: lytic or lysogenic [22]. Virulent or lytic phages infect their host exclusively by the lytic lifecycle, where phage infection ultimately results in lysis of the host bacterium and release of progeny phages particles. However, temperate or lysogenic phages can also follow a lysogenic lifecycle, where infection leads to the integration of the phage genome into that of the host forming a prophage. In this manner, the phage can exist as part of the host genome and replicate as a single unit without destruction of the cell. In this lifestyle, lytic genes are repressed, while other genes also influencing the host fitness can be expressed. Expression of such genes can alter host phenotypes relating to pathogenicity, prophage immunity, biofilm formation and stress response [23,24]. Additionally, prophages have been implicated as agents of horizontal gene transfer between bacteria by means of transduction [25].

We currently have a limited understanding of how phages may influence *E. lenta* and other members of the class Coriobacteriaa in the gut microbiome. To date (January 2020), GenBank only contains a single genome of a phage confirmed to infect *E. lenta* (*Eggerthella* phage PMBT5, accession no: MH626557), with an additional thirteen phage genomes identified from human gut viromes using *E. lenta* derived CRISPR spacer sequences [26]. In this study, we selectively isolated several novel strains of *E. lenta* and compared their genomes to those publicly available. We characterised the prophages infecting this bacterium and showed that they represent new viral genera not described in current literature. The characterisation of their genomes gives us insights into how Actinobacteria phages prey on and potentially influence host fitness.

## 2. Materials and Methods

### 2.1. Bacterial Growth Requirements

*E. lenta* strains were cultured using BHI++ broth or BHI++ agar (1% *w*/*v* agar) [27], with incubation at 37 °C under anaerobic conditions. All *E. lenta* strains were stocked in BHI++ containing 40% (*v*/*v*) glycerol and stored at −80 °C. Details of *E. lenta* cultures utilised in this study are provided in Supplementary Information S1, Table S1.

### 2.2. Antibiotic Resistance Screening

The minimum inhibitory concentration (MIC) values of ceftriaxone (Sigma-Aldrich, St. Louis, MO, USA) and cefotaxime (Sigma-Aldrich, St. Louis, MO, USA) were determined using a broth dilution method (two-fold). A 48 h culture was inoculated at 1% (*v*/*v*) in a 96 well plate and incubated at 37 °C for 72 h in anaerobic conditions with absorbance values taken with a microplate reader (Thermo Fisher, Waltham, MA, USA). The MIC was defined as the concentration of antibiotics that resulted in no appreciable bacterial growth.

### 2.3. E. lenta Isolation from Human Faecal Samples

Human faecal samples were collected from ten adult volunteers who were enrolled by APC Microbiome Ireland as part of a study examining the human gut virome, according to study protocol APC055 approved by the Cork Research Ethics Committee (CREC). Faecal samples were transported to the laboratory, aliquoted and frozen at −80 °C within 2–3 h from voiding. Look to Supplementary Information S1, Table S2 for isolate source details for *E. lenta* isolates. *E. lenta* isolation from faeces was performed using BHI++ agar (1% *w*/*v* agar) and BHI++ overlay (0.25% *w*/*v* agarose) supplemented with ceftriaxone (20 µg/mL) and cefotaxime (2 µg/mL). Faecal samples were resuspended in PBS buffer at 0.5 g/mL, serially diluted and added to overlay and poured to agar, with subsequent incubation in anaerobic conditions at 37 °C for 72 h. Presumptive *E. lenta* colonies (those with brown/yellow pigmentation) were examined by light microscopy utilising Gram staining and a species-specific colony PCR using MyTaq Red Mix (Bioline, London, UK) using primers previously described (Supplementary Information S1, Table S3) [10]. A total of eight confirmed *E. lenta* strains and seven additional unidentified colonies were selected for further analysis utilising genome sequencing.

### 2.4. Bacterial Genomic DNA Extraction and Genome Sequencing

Genomic DNA was isolated using the GenElute™ Bacterial Genomic DNA Kit (Sigma-Aldrich, St. Louis, MO, USA). DNA was then quantified using the Qubit broad range assay (ThermoFisher Scientific, Waltham, MA, USA) before standardisation for paired-end Nextera XT library preparation (Illumina, San Diego, CA, USA). Libraries were inspected with the TapeStation 4200 system (Agilent, Santa Clara, CA, USA) using the High Sensitivity D1000 ScreenTape Assay before sequencing with an Illumina NovaSeq platform (Illumina Inc., San Diego, CA, USA). Illumina reads were adapter trimmed using Trimmomatic 0.30 [28]. De novo assembly was performed on samples using SPAdes (v3.7) with the removal of contigs <500 bp among draft genomes. QUAST (v.5.0.2) was used to assess assembly quality [29,30]. Genome completeness and contamination were determined using CheckM (v1.0.18) [31].

Long-read genomic DNA libraries were prepared with an Oxford Nanopore SQK-LSK109 kit with Native Barcoding EXP-NBD104 (Oxford Nanopore Technologies, Oxford, UK). Barcoded samples were pooled together into a single sequencing library and loaded in an FLO-MIN111 (R.10.3) flow cell in a MinION (Oxford Nanopore Technologies). Genome assembly of circular genomes was conducted with Unicycler (v0.4.8) using short and long reads obtained from Illumina and Nanopore sequencing, respectively [32]. Short and long read sequence coverage was determined with Bowtie2 (v2.3.4.1) and minimap2 (v2.17), respectively, with Samtools (v1.7) [33,34,35].

### 2.5. Bioinformatic Analysis of Bacterial Genomes

Dot plots illustrating genome alignment were created using MUMmer [36]. Average nucleotide identity was calculated using Pyan (v0.2.20) (ANIm) [37]. Genome annotation was conducted with Prokka v1.11 [38] utilising a protein database derived from strains DSM2243 and C592 obtained from the NCBI nucleotide database (https://www.ncbi.nlm.nih.gov/nuccore/, accessed on 2 March 2020), and all publicly available genomes sequences were obtained from the NCBI SRA database (https://www.ncbi.nlm.nih.gov/sra/, accessed on 2 March 2020). Pangenome analysis was conducted with Proteinortho (identity = 30%, coverage = 70%) [39]. Accumulation curves for pan and core genome were calculated by Proteinotho_curves (https://codeload.github.com/isabelschober/proteinortho_curves/, accessed on 2 December 2021). PanGP (v1.0.1) using the TR algorithm (sample size 500, sample repeat 10) was used to assess the openness of the pangenome (y = A_pan_x^Bpan^ + C_pan_, where y is pan-genome size, x the genome number and A_pan_, B_pan_ and C_pan_ fitting parameters) with findings were interpreted by Heap’s law [40,41]. Determination of the function of bacterial orthologous groups was performed by RPS-BLAST (v2.2.15) against the COG database (2 January 2010) [42,43]. Phylogenetic analysis based on bacterial core genes was generated with ROARY(v3.13.0) and PRANK(v170427), while the construction of a phylogenetic tree was performed using FastTree [44,45,46].

### 2.6. Prophage Features Identification, Phylogenetic and Protein Functional Analysis

Prophages were identified by alignment of different *E. lenta* genomes with either strains DSM2243 or C592 using Mauve using progressive alignment [47]. A heatmap based on nucleotide homology was calculated using Gegenees (v3.1.0) utilising BLASTn, with accurate parameters [48]. Phylogenetic trees based on prophages proteomes was generated using VICTOR [49]. Phylogenetic analysis of proteins was conducted using MEGAX [50], utilising MUSCLE [51] for sequence alignment with the construction of phylograms using the maximum likelihood method based on the Jones–Thorthon model [52]. For a graphic illustration of phylograms, ITOL was utilised [53]. Gene sharing networks of phages was conducted using vConTACT2 (v0.9.13) (pcs-mode MLC, vcs-mode ClusterONE), with resulting networks visualized with Cytoscape (v3.8.2) [54,55]. Phage genomes used for the vConTACT2 database were obtained from NCBI (May 2020) with proteins predicted with prodigal (v2.6.3) [56]. VIPtree (v2) was also utilised to taxonomically place prophages [57]. Prophage related orthologous groups were further annotated using the PHASTER prophage-virus database (August 2019), InterProScan5 and the Prokaryotic Virus Orthologous Groups (pVOGs) database (downloaded December 2020) using hmmscan (v.3.1b2) [58,59,60,61,62]. Prophage virion protein and morphology were determined using VirFam [63]. When necessary, proteins were manually annotated using Hhpred [64]. For the analysis of host CRISPR spacers targeting *E. lenta* prophages MINced (v0.4.2) was used to extract *E. lenta* spacers (https://github.com/ctSkennerton/minced (accessed on 2 November 2020)), while BLASTn (-task BLASTn-short) was used to identify protospacer among prophage genomes possessing no more than 3 misaligned bases with bitscore being ≥44.1 [65]. To compare prophage genome coverage with that of the host, depth of sequence coverage was determined by aligning reads to the entire host genome and the prophage utilising Bowtie2 v2.3.4.1 and Samtools v1.7 [34,35].

### 2.7. Demonstration of DGR Functionality of Prophage DSM2243phi4

BHI++ broth was used for the growth of DSM2243. All fermentations were performed in a continuous format for four days with controlled conditions in a final volume of 200 mL and using myControl Minibio-500 mL systems (Applikon Biotechnology, JG Delft, The Netherlands). The dissolved oxygen level was maintained below 0.1% by sparging with an anaerobic gas mix (80% (*v*/*v*) N2, 10% (*v*/*v*) CO2, 10% (*v*/*v*) H2). The medium was kept at 37 °C with constant stirring of 50 rpm and a controlled pH of 6.8. The vessel was inoculated with 2 mL of DSM2243 for 24 h in batch. After that, fresh broth was pumped to the vessels at a rate of 400 mL/day. The culture was then plated in a manner that allowed the isolation of randomly selected colonies, which were subsequently prepared for genome sequence in the same manner as previously mentioned.

### 2.8. Detection of Circularised Prophage Genomes among Host Strain Cells

PCR verifications were used to detect circularised prophage genomes. To determine the presence of prophage predicted *attP* sites primers were utilised that were specific boundaries of these genome loci. MyTaq Red Mix (Bioline, London, UK) was utilised for PCR performed on *E. lenta* cells resuspended in phosphate buffer saline (PBS) buffer. Details of primers and annealing temperatures for these PCRs are provided in Supplementary Information S1, Table S2. Subsequently, the resulting PCR products were sent for sanger sequencing and aligned to the respective prophage genome.

### 2.9. Detection of Virions in the Supernatant of Strain DSM2243

The supernatant obtained from a pelleted culture of DSM2243 was treated with DNase I (6 µg/mL) and RNase A (3.34 µg/mL) at 37 °C for 30 min, with subsequent treatment with 10% SDS and proteinase K (60 µg/mL) at 55 °C for 30 min. DNA extraction was then performed with phenol: chloroform: isoamyl alcohol (25:24:1 *v*/*v*) and chloroform: isoamyl alcohol (24:1 *v*/*v*). DNA precipitation was conducted with 0.3 M sodium acetate (pH 5.2) and isopropanol (50% *v*/*v*) and subsequent DNA clean-up with Zymogen clean/concentration columns (Zymo Research, CA, USA). Library preparation using the Nexteria XT kit and genome sequencing was conducted in the same manner as previously mentioned, with sequence coverage being determined with Bowtie2 and Samtools, using both forward and reverse sequence reads.

### 2.10. Data Processing and Visualisation

Data manipulation and graphic illustration were performed using the R environment (https://www.r-project.org, accessed on 2 December 2021) with the following packages: reshape, ggpolt2, tidyverse, heatmap.2, and complex heatmap.

### 2.11. DDBJ/ENA/GenBank Submission Details

For accession numbers for genomes and plasmids of *E. lenta* isolated in this study look to Table 1. Please contact authors to obtain identified prophage genome sequences discussed in the study.

## 3. Results

### 3.1. Isolation of E. lenta from Human Faecal Samples

For *E. lenta*, as well as most species of the order Coriobacteriia, there is limited information regarding growth media that can be used for their selective isolation [66]. Most well-described isolates of this bacterium have been obtained using non-selective media or procedures only based on inherent metabolic features (e.g., oxidation of digoxin or conversion of lignans) [67,68,69]. In this study, we devised a selective agar composition based on BHI++ media as described by Bisanz et al. [27], supplemented with the broad-spectrum antibiotics cephalosporins ceftriaxone and cefotaxime as the bacterium has been reported to be highly resistant to both agents (we term this media as Elen-BHI++, hereafter) [18,70]. We first verified that this antibiotic resistance is present among several strains in our possession [*n* = 7] with a median MIC of >156 µg/mL for both ceftriaxone and cefotaxime (Supplementary S1, Table S4).

For *E. lenta* strain isolation, human faeces from ten different individuals were diluted and plated onto Elen-BHI++ agar. When serial diluted human faeces was plated onto this selective agar and subsequently incubated under optimal conditions, it resulted in the formation of a limited number of colony morphologies enabling the identification of those presumptive to be *E. lenta*. Those colonies found to possess a distinct dark yellow/brown pigmentation were identified as this bacterium (Supplementary S2, Figure S1). Strains of this species have been reported to produce this pigmentation on BHI++ media, a phenomenon that is suspected to be related to its predicted ability to produce carotenoid compounds [27]. It was also observed that this colony pigmentation was found more consistently when the bacterium was grown using double overlays utilising soft agarose of Elen-BHII++ medium. Eight of the ten faecal samples yielded presumptive *E. lenta* colonies and these were subsequently confirmed by species-specific colony PCR. Enumeration of these colonies ranged from 1.3 × 10^5^ to 4.2 × 10^6^ CFU/g with a median of 4.2 × 10^5^ CFU/g [*n* = 7] of faeces. Selected *E. lenta* isolates were confirmed as Gram-positive rods under light microscopy, with cells appearing either singularly or in long chains (Supplementary S2. Figure S2). Other dominant colony morphologies identified to grow on Elen-BHI++ agar (white colonies of varying size) were identified as *Bacteroides fragilis* or *Bacteroides uniformis*.

### 3.2. Genomes of E. lenta Isolates

**Figure 1:**
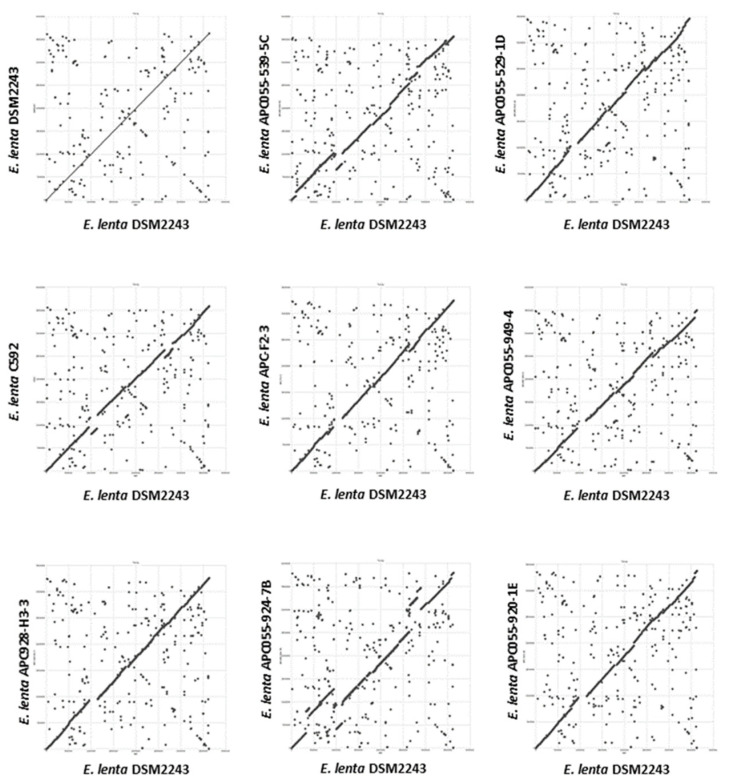
Dot plot illustrating whole genome alignment (MUMmer) of seven *E. lenta* strains isolated in this study (denoted with the APC prefix) and those publicly available (C592 and DSM2243) with the genome of the type strain DSM2243.

Genome assembly was performed using short and long-reads to obtain high-quality complete genomes for seven *E. lenta* isolates obtained in this study (Supplementary Information 1, Tables S1 and S2). Average sequence coverage for short and long reads being 1129 and 31-fold, respectively. Sequenced isolates were found to have an average genome size of 3.34 ± 0.246 mbp with a high GC content of 63.54 ± 0.95% (mean ± SD); these values are within ranges typically reported for this species [71]. Dot plot alignments were performed with the seven fully-sequenced genomes of *E. lenta* obtained in this study (APC055-539-5C, APC055-529-1D, APC055-949-4, APC055-928-H3-3, APC055-924-7B, APC055-920-1E and APC-F2-3) with those publicly available (C592 and DSM2243). Based on the obtained dot plots, variability is observed across members of *E. lenta* species, with general conservation of genome synteny. Occasional break points are observed at multiple chromosomal locations across the nine compared genomes, indicative of insertions/deletions events that occurred within the species (Figure 1).

Furthermore, cryptic plasmids were obtained with the genomes of APC055-529-1D (single plasmid) and APC-F2-3 (two plasmids). These plasmids ranged from 3086 to 3844 bp in size with a GC content of 58 ± 1% and containing between 5 and 6 CDS, with each plasmid possessing a gene that could be identified as a plasmid replication protein (identified to possess a Rep_2 domain [PF01719] or a distantly related structural homolog). Similar elements have previously been described to be found among this species [72]. The sequence depth of these plasmids was found to be 5 to 33-fold greater than that of the host genome.

These genomes were compared to 50 non-redundant genomes of *E. lenta* obtained from public repositories, comprised of two complete and 48 high-quality draft sequences with a low number of contigs (median of 58 (range 13–465)) (Supplementary Information S1, Table S1). Based on our comparison, *E. lenta* isolates were found to possess an average nucleotide identity (ANI) of ≥97%, with genomes of this species sharing an ANI of ≈88% with the type of strain of *Eggerthella sinesis*, the closest related species to *E. lenta* currently defined in the literature (Supplementary Information S2, Figure S3).

### 3.3. Comparative Analysis of E. lenta Isolates

**Figure 2:**
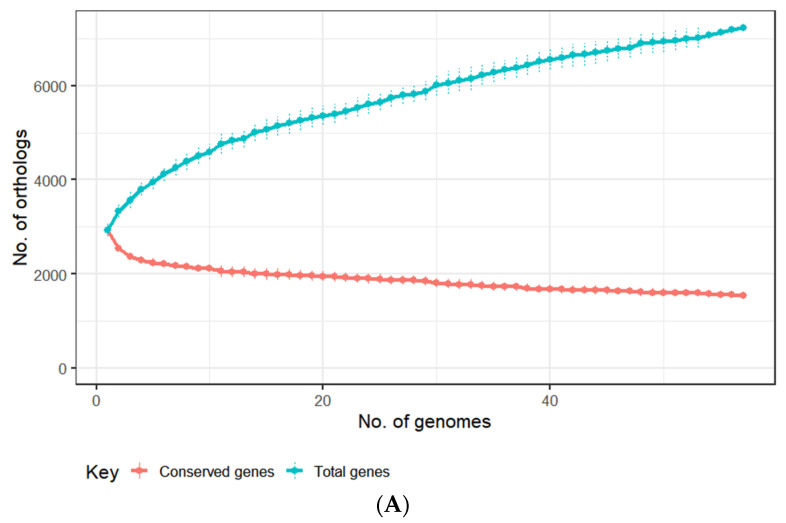

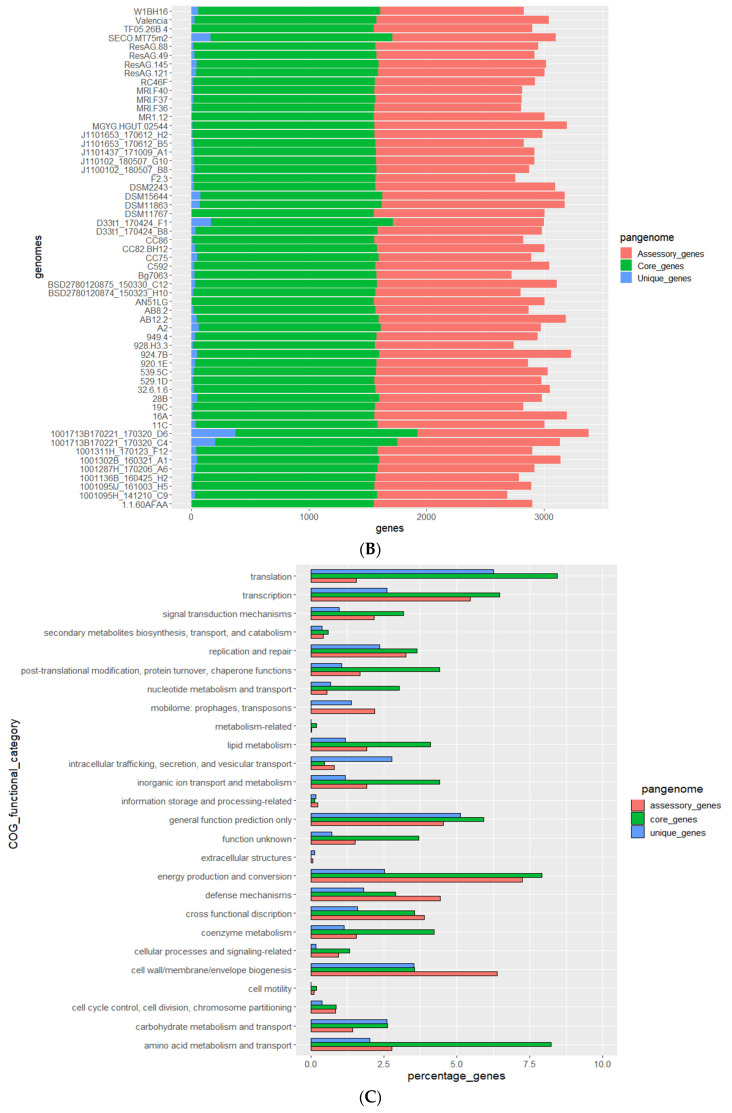
(**A**) Distribution of genes in the pangenome among each of the examined 57 genomes of cultivated *E. lenta* isolates. (**B**) Analysis of dereplicated genomes of *E. lenta* (*n* = 57) shows the genus possesses a pan and core genome of 8273 and 1425 genes, respectively. (**C**) OGs of the pangenome (core, accessory and unique genes) of 57 *E. lenta* isolates and their functional assignment using the COG database, the fraction of OGs assigned no function is excluded from illustration.

To facilitate the identification of prophage sequences associated with this species, a pangenome analysis was performed with the *E. lenta* genomes (*n* = 57) based on the clustering of their predicted proteins into orthologous groups (OGs). These genomes were identified to contain an average of 2933 ± 115 proteins, which could be placed into a total pan-genome of 7235 OGs (identity = 30%, coverage = 70%) with a core genome of 1547 Ogs (Figure 2A). This analysis allowed the categorisation of the genes of *E. lenta* into three categories: core (genes shared among all isolates), accessory (genes shared among some isolates) and unique (genes unique to a particular isolate). Our genome comparison established that the core genome represents approximately 53% of the number of genes found on an average sized *E. lenta* genome (Figure 2B). This is within the range of that reported for other bacterial species associated with the human gastrointestinal tract, with core genomes ranging from 44% to 61% for *Escherichia coli* and *Bifidobacterium* *longum*, respectively [73,74]. Based on our comparative analysis the *E. lenta* pan-genome was found to be open (when interpreted using Heap’s law), implying that the size of the pan-genome will tend to increase with the analysis of additional genomes [41]. Accordingly, each genome within this dataset was found to provide a median of 23 unique genes (range 5 to 373) not shared among other genomes (Figure 2B).

The COG (cluster of orthologous groups) database is constituted of proteins whose functions are assumed to be derived from ancestral proteins with similar or identical functions and is a popular tool for functional classification of protein function [42]. The RPS-BLAST alignments against the COG database allowed COG assignment of 58% of OGs of *E. lenta*. OGs identified are involved in the metabolism of carbohydrates, amino acids, lipids, and secondary metabolites, which can be found to be part of the core genome. Additionally, such OGs were found throughout accessory and unique genes (Figure 2C). Our analysis highlights that there is a core set of metabolic capabilities associated with all species but there are also strain-specific differences across isolates. It has already been shown that certain *E. lenta* strains vary in their capacity to act on digoxin and lignans [12]. In accordance with previous observations, our analysis identified strain differences in genes implicated in the inactivation of drugs such as digoxin (present in 28/57 genomes) and dopamine (present in 55/57 genomes), as well as the activation of phytochemicals such as lignans (present in 45/57 genomes). Furthermore, the examination of genes implicated in antibiotic resistance showed that beta-lactamases are a highly prevalent feature of this species with the identification of at least two varieties (PF13354, PF00144) of the enzyme among 56/57 and 55/57 genomes, respectively (Supplementary Information S2, Figure S4). Notably, this result contrasts with previous reports suggesting that this bacterium does not produce beta-lactamases [70,75]. The possession of these antibiotic resistance genes likely explain the high resistance of this species to ceftriaxone and cefotaxime found in this study and others [18,70]. Our analysis also showed that gene products associate with mobile genomic elements such as transposons and prophages are entirely associated with accessory and unique genes making up 2.2% (77 OGs) and 1.4% (33 OGs), respectively (Figure 2C). This result indicates that these gene products are not shared among all isolates, with some constituting unique features present in particular isolates.

### 3.4. Identification and Diversity of Prophages

**Figure 3:**
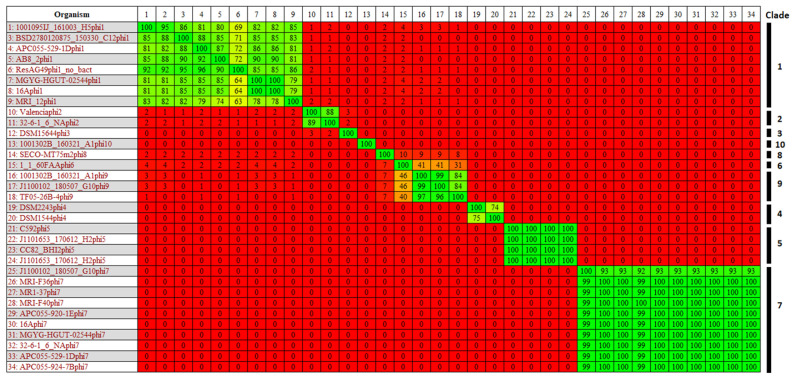
BLASTn heat map generated using Gegenees with accurate parameters—fragment length: 200 bp; step size: 100 bp; threshold: 0%. The map includes 33 identified prophage genomes of *E. lenta*, members of the ten different prophage clades are highlighted.

Screening of the pangenome of 57 *E. lenta* genomes for OGs encoding hallmark phage proteins (terminase, major head and portal protein) resulted in the identification of prophage-like elements in the genomes of 26 strains. In total, 33 prophages were identified with some strains found to possess up to two distinct prophages per genome (Table 2). Utilising progressive Mauve for sequence alignment of these *E. lenta* genomes with isolates DSM2243 or C592 (strains with publicly available complete genomes sequences) allowed determination of the approximate location of prophage termini. The genome sizes of complete prophages (those with approximate genome termini determined) were found to vary from 32 to 42 kb, with GC contents typically lower than that of their host ranging from 58 to 67%. In five cases, we could not identify prophage termini due to the incompleteness of host draft sequences. Gegenees derived BLASTn analysis allowed the clustering of these prophages in ten distinct clades, each sharing an identity of >60% across the whole prophage sequence at the nucleotide level, with a higher level of inter-clade relatedness indicated with a homology of >40% at nucleotide level between clades 6 and 9 (Figure 3). These prophage clades were further confirmed by sequence analysis of their proteomes and phylogenetic analysis using VICTOR (Supplementary Information S2, Figure S5). Of note, the most populated clades were 1 and 7, collectively representing more than half of the prophages identified.

The prediction of potential integration sites (*attB*) was performed using genome alignments, and we were able to predict an *attB* site for 26 of the 33 prophages (Table 3). Prophages of four clades (1, 2, 3, 7) integrate at transfer RNA (tRNA) genes for arginine, alanine, serine and leucine. Similar attachment sites have been described for prophages infecting two other Actinobacteria species, *Bifidobacterium* and *Mycobacterium* [76,77,78]. Additionally, prophages of clade 5 appear to integrate into the coding sequence for a hypothetical protein with predicted DNA binding activity (PF01381). Unfortunately, no putative attachment side could be assigned using this methodology for the remaining five clades. Additional confirmation for the attachment of clade 1 was determined by detecting direct terminal repeats in six of the seven representatives for which approximate prophage sequence boundaries were known. The terminal repeats look to represent the *attP* sites for these prophages and possess sequence homology to tRNA genes when aligned to the genome of the type of strain (Table 3)

### 3.5. Gene Content of Prophages and Possible Impact on Host Infection

**Figure 4:**
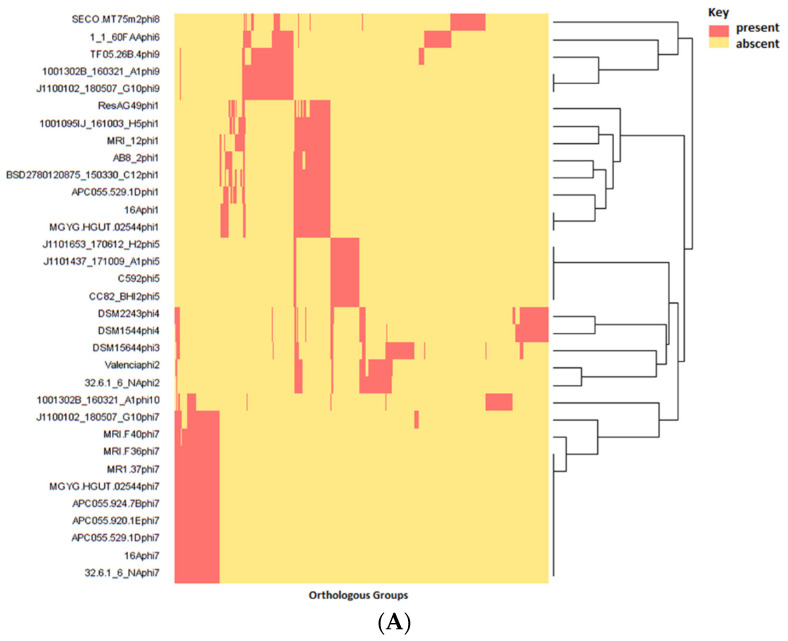

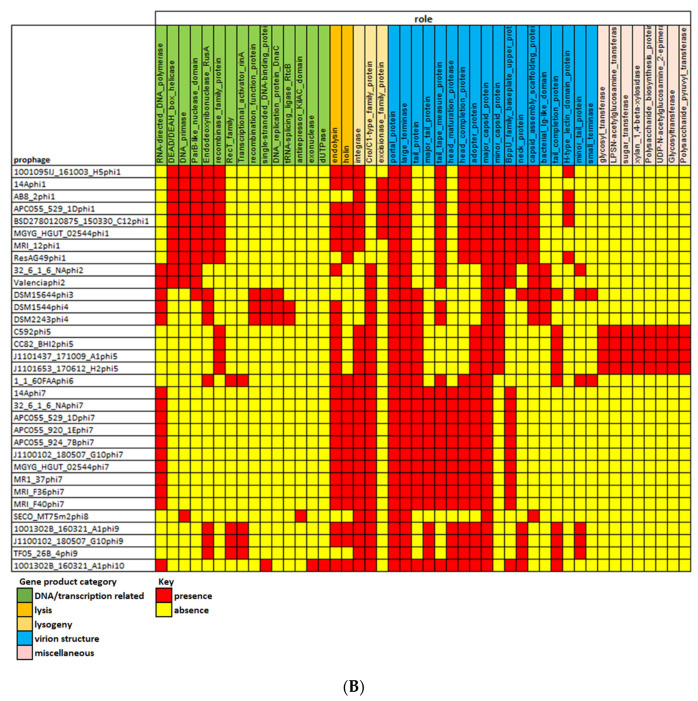
(**A**) Heatmap showing the distribution (presence–absence of OGs among prophages of *E. lenta*. (**B**) Heatmap illustrating genes detected among *E. lenta* prophages found with a particular function.

The number of ORFs per prophage ranges from 35 to 64, and these can be assigned to 418 OGs (identity = 30%, coverage = 70%), representing approximately 5.8% of the total pangenome of the host species. Prophage proteins are highly diverse, with limited homology between prophage clades (Figure 4A). Of the 418 OGs, only 98 were found to be shared across clades (such OGs being shared among a median of two clades (range 2–5)). Notably, only 37% of these OGs could be given a functional assignment. At the level of protein function, overlap could be observed among different OGs, an observation that resulted in the designation of multiple OGs among prophages implicated with similar function (Supplementary Information S2, Figure S6). For this reason, our annotation efforts classified prophage OGs into five major functional categories of virion assembly, lysogeny, host lysis, DNA replication and maintenance related, transcription and accessory (Figure 4B).

Among proteins implicated in virion structure those involved in DNA packaging (major capsid, large terminase, portal protein) were present in all representatives as would be expected for genes encoding core phage features. Phylogenetic analysis of the large terminase indicates that the DNA packaging strategy of prophages clades 1 to 6 is a headful system related to *Bacillus* phage SPP1, while that of clades 7 to 10 use a cos-type system related to *Bacillus* phage phi105 and *Lactococcus* phage phiLC3 (Supplementary Information S2, Figure S7) [79,80,81]. Virion tail related proteins were also identified among the most frequently found functions, encoding for the tail tape measure (6/10 clades), tail completion (5/10 clades) and baseplate upper protein (4/10 clades), in accord with a tailed virion morphology. In fact, the structural proteins (major capsid, adopter, head completion and neck protein) are all consistent with a *Siphoviridae*-like morphology (Supplementary Information S1, Table S5). Of note, predicted structural proteins with Ig-like domains were found among prophages of clades, 2, 3 and 4. It has been previously shown that these domains are implicated in phage adherence to glycan residues present in mucin glycoprotein, often associated with mucosal surfaces such as the intestinal gut wall [82]. The presence of such domains on the virions of phage infecting *E. lenta* would likely be advantageous as the bacterium has been shown to possess high adherence to gut epithelial cells in cell culture, indicating the bacterium strongly associates with the intestinal gut wall [83].

**Figure 5:**
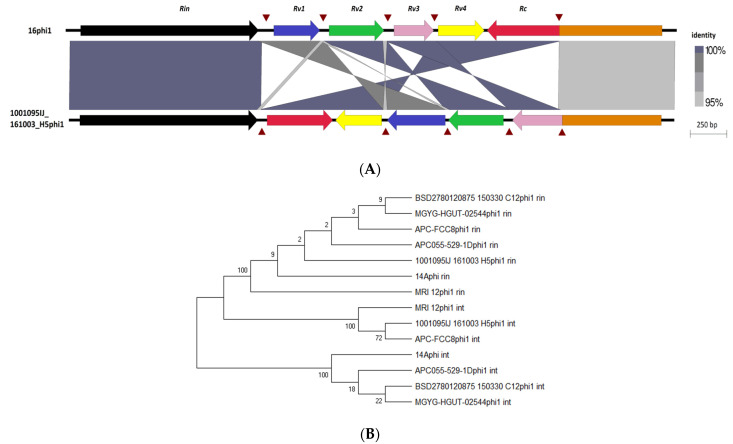
Characterisation of Rin shufflon among *E. lenta* prophages. (**A**) Locus map showing the alignment of the Rin shufflon among prophages 16 phi1 and 1001095IJ_161003_H5phi1, genes (arrows) relative to the direction of transcription and labelled accordingly to function. Brown triangles indicate repeat regions and shaded regions represent the degree of sequence homology in BLASTn alignments. (**B**) Unrooted maximum likelihood tree of invertases and intergrases among *E. lenta* clade 1 prophages.

A recent study conducted on prophages of another Actinobacteria genus identified that phages infecting *Bifidobacterium* possess the so-called *Rin* system, with an RBP-locus tyrosine-family DNA invertase, implicated in conferring diversity in host range specificity [76]. Similarly to what was observed for *Bifidobacterium*, alignment of *E. lenta* prophages of clade 1 showed high gene synteny except for one location directly downstream of a tyrosine recombinase (*Rin*) (PS51898) where several small tandemly oriented genes (*Rv*) are also located, often encoding for small proteins with H-type lectin domain (Figure 3). These domains are typically involved in carbohydrate binding and can be found associated with phage RCB proteins, suggesting that genes of this prophage clade are implicated in host specificity [62]. These *Rv* genes can have homology with the C-terminus of a much larger gene (*Rc*) located directly downstream, thus indicating that gene recombination and shuffling occurs in these loci. Furthermore, we identified a short asymmetric 8 bp repeat (5′-ttccgtat-3′) upstream and downstream of each *Rv* gene. This repeat sequence can be found inverted downstream of the *Rc* gene just after a stop codon, as well as located within the gene itself. These are expected to be the crossover sites (*rix*) that allow inversion to occur. This repeat sequence does not commonly appear in other regions of this prophage clade. Like the system found in *Bifodobacterium* prophages, *Rv* genes possess limited homology to each other (Figure 5A), and the tyrosine recombinase is distinct from the tyrosine integrase, which possesses similar domain architecture and is located at the opposite wing of the genome of *E. lenta* clade 1 prophages (Figure 5B).

As would be expected for prophages, genes typically associated with lysogeny could be found among most prophage clades, such as an integrase (7/10 clades) responsible for the insertion of a prophage into the host genome, all identified as tyrosine integrases (IPR002104). We also found genes containing the Cro/C1-type repressor family domain (9/10 clades), a domain associated with Cro and C1 proteins of coliphage Lambda, which act as gene repressors for the regulation of lysogeny. Lysis related genes were also found with proteins implicated in peptidoglycan degradation (8/10 clades). These possess varying enzymatic activities, with amidase being the most common (5/10 clades), followed by glycosidase hydrolase (2/10) and CHAP domains (1/10 clades). Furthermore, these proteins could be found to possess cell wall binding domains of varying types (Cholin Binding, SH3-like, PGBDSf, CW_7 and LysM). Most of these identified peptidoglycan degrading proteins are expected to play a role as endolysins due to their proximity to holin encoding genes. However, unlike genes implicated in virion structure, lysogeny and lysis, less success was achieved in the identification of genes implicated in DNA replication. Of note, this difficulty in identifying genes associated with DNA replication has also been observed among *Bifidobacterium* prophages [77], most probably indicating a current lack of reference genes in public databases.

**Figure 6:**
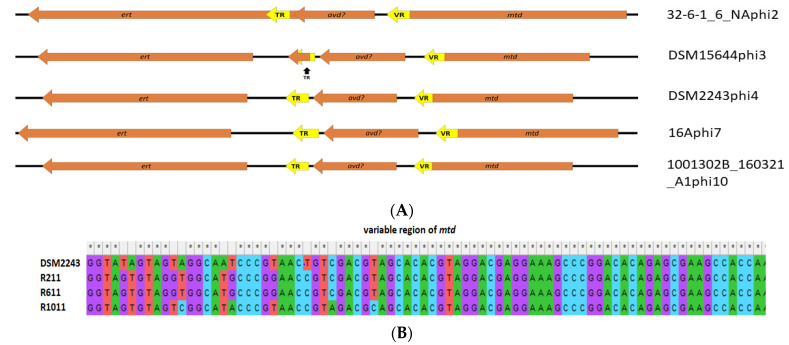
(**A**) Locus map of the architecture of the DGR element found among different prophage types of *E. lenta*, showing genes (arrows in orange) and sequence repeat regions (arrows/arrow segments in yellow) relative to the direction of transcription and labelled accordingly to predicted function. (**B**) Alignment of a VR segment of the *mtd* gene forming part of the DGR element of a clade 4 prophage associated with strain DSM2243, to isolates of the bacterium (R211, R611 and R1011) grown after fermentation in a chemostat (24 h as batch, then 72 h as continuous) illustrating locations of nucleotide substitutions.

However, one interesting finding among this category of gene products was the detection of ORFs with gene products predicted to encode RNA-dependent DNA polymerase (IPR000477) that we term *ert* (*Eggerthella* reverse transcriptase) among prophage clades 2, 3, 4, 7 and 10, which appear to form a diversity generating retro (DGR) element. Among *Bordetella* temperate phages (BPP-1, BIP-1 and BMP-1), RNA dependent DNA polymerase has been demonstrated to act as part of a system that causes nucleotide substitutions with genes for virion proteins involved with host specificity, resulting in phase variation [84]. It is likely that a similar system exists among *E. lenta* prophages. Members of clade 7 prophages were found to be highly related at the nucleotide level (Figure 3 apart from the 3′ termini at the variable region (VR) of the *mtd* (major tropism determinant) gene located downstream of genes implicated in virion structural proteins. VR is a tandem repeat (TR) of a region located upstream of the 5 termini of *ert* that is 130 bp in size (BLASTn identity >90%). The architecture of this system in clade 7 *Eggerthella* prophages resembles that of the previously discussed *Bordetella* phages, even with a possible equivalent of the *avd* (accessory variability determinant) gene situated between the TR region and the *mtd* gene, the product of which complexes with Ert (the equivalent of Brt among *Bordetella* phages) and facilitates the DGR process [85]. The *brt* gene product utilising the TR region causes site-specific mutagenesis of the VR region [86]. This architecture of DGR with *ert*, *mtd*, predicted *avd* genes as well as the TR and VR regions, are similar among prophages in clades 3, 4 and 10. However, a slightly different configuration is observed among prophages of clade 2 (Figure 6A). Among these prophages, the TR region lies within the *ert* gene with this region being 123 bp long, where once again the VR region (BLASTn identity >90% to TR) is positioned towards the 5′ end of the *mtd* gene. Alignment of the VR regions between members of prophage clade 2 shows a high density of nucleotide substitutions. We could not assign a role to the *mtd gene* among these prophages, but it is suspected to play a role in virion structure due to their proximity to ORFs implicated with such function among these prophages. For *Bordetella* phage BPP-1, *mtd* has been identified to encode a protein that forms part of the tail fibre of the virion of this phage [87]. We were able to obtain evidence for the functionality of the DGR element of a clade 4 prophage associated with *E. lenta* strain DSM2243 (type strain). After fermentation in a chemostat (24 h as a batch, then 72 h as continuous), we isolated randomly selected colonies and subjected them to genome sequencing (average coverage >300). Analysis of the VR region of the *mtd* gene among such isolates shows nucleotide substitution at 10 different sites, enabling DSM2243phi4 to explore a sequence space of potentially up to 10^6^ unique variants concerning this locus (Figure 6B). The finding also indicates that the system is active while the prophage remains in its lysogenic state.

Prophages among several bacterial species can also possess genes that impact host fitness. This also appears to be the case for *E. lenta* in which prophages of clade 5 possessing an operon of up to six genes implicated in exopolysaccharide biosynthesis (or possibly capsular polysaccharides). Three gene products can be broadly classed as polysaccharide transferases, one of which possesses the polysaccharide pyruvyl transferase domain (IPR007345) associated with WcaK of *Escherichia coli* implicated in the formation of colanic acid. Another of the proteins possess the domain UDP-N-acetylglucosamine 2-epimerase WecB-like (IPR029767) that is related to WecB in *Enterobacteriaceae*, implicated in the formation of a surface antigen polysaccharide.

Other interesting accessory genes include a toxin–antitoxin system in prophage clade 6 that could play a role to ensure the retention of prophage in daughter cells, as seen with phage N15 of *Escherichia coli* [88]. Furthermore, the possession of an abortive infection (ABI) system protein (IPR011664) is present in members of prophage clades 5 and 7. It is tempting to speculate if such proteins may play a role in preventing infection of the host by competing prophages.

### 3.6. Taxonomic Placement of Prophages

**Figure 7:**
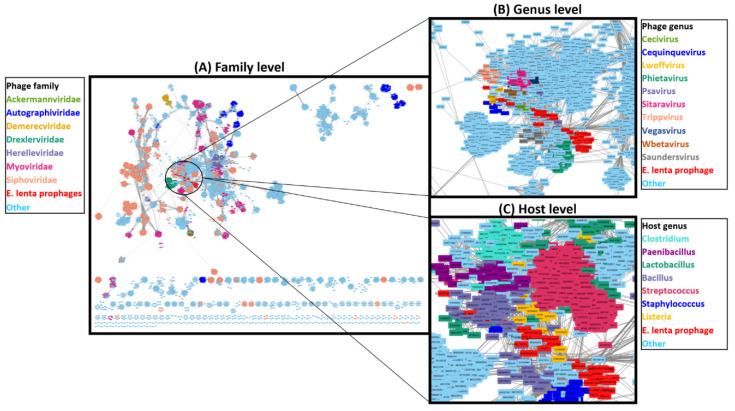
Network-based analysis of shared protein clusters with 33 *E. lenta* prophages with 12,892 phages visualised with Cytoscape. Each node represents a phage genome, lines (edges) represent the strength of connectivity (edge weight) between genomes. (**A**) Overview of network with nodes coloured to highlight family to which a phage belongs and a close-in view of the network of where *E. lenta* prophages are situated with nodes coloured to highlight (**B**) phage genus or (**C**) or host bacterium genus.

To better understand the taxonomic position of *E. lenta* prophages relative to currently available phage genomes, we conducted a network-based analysis of shared protein clusters using vConTACT2 and a database of 12,892 phage genomes (Figure 7). The analysis placed the 33 prophages into six viral clusters (Supplementary Information S2, Table S6), which approximates to genus level ranking in relation to ICTV classifications [54]. However, under-sampling with respect to the number of genomes available to representatives of each prophage clade has likely resulted in this placement, as the diversity of each prophage clade appears sufficient for genus designation (shared nucleotide sequence similarity >50%) [89]. These clusters were positioned within a complex network comprising of phages belonging to *Siphoviridae* containing several defined phage genera with bacterial hosts mostly situated among the phylum Firmicutes, but also some representatives of Actinobacteria. Ten phage genera could be identified to be situated within this cluster, all infecting members of the Firmicutes. The closest defined genus that could be associated with the *E. lenta* prophages was that of *Cequinduevirus* (average edge weight 5.20) to prophage clade 9. Moreover, analysis with VIPtree further indicates a distant relationship between *E. lenta* prophages and *Cequinduevirus* (Supplementary Information S2, Figure S8). This genus comprises phages infecting the genus *Lactobacillus*—type phage *Lactobacillus* phage c5. These phages possess a *Siphoviridae* morphology and are suspected of having evolved from a lineage of phage that was once temperate due to their possession of proteins similar to Cro-like repressors, but now lack genes encoding other proteins necessary for this phage lifestyle [90]. Genome alignment of clade 9 prophages to those of *Cequinduevirus* shows that there is homology between several proteins implicated with virion capsid formation and DNA packaging (Supplementary Information S2, Figure S9). This suggests that these phages likely utilise a similar DNA packaging strategy as *Lactobacillus* phage C5, which utilises a cos-type system [90].

### 3.7. Prophages and the CRISPR/cas System

*E. lenta* possesses a CRISPR/cas system of type I-C subgroup that has been demonstrated to be functional and is understood to target mobile genetic elements such as plasmids and prophages [26]. However, information on the CRISPR/cas system and its impact on prophages of *E. lenta* has yet to be described. Among the 58 genomes of *E. lenta* examined in this study, 44 were identified to possess a spacer array. These arrays were identified to possess between 13 to 104 spacers (median 52) with a median size of 34 nucleotides. In total, 2283 spacers (555 unique) were identified among their genomes, with 283 spacers (46 unique) found to target prophages of this species.

Only two of the 44 *E. lenta* genomes with identified CRISPR spaces did not target the prophages identified in this study. The remaining genomes were found to have arrays that harbour at least one spacer, with a maximum of three nucleotide miss matches, that could target a representative of a single prophage clade (7/44) (Figure 7), with others targeting up to two (7/44), three (27/44) or even four different prophage clades (1/44). Our analysis also shows that a single *E. lenta* genome can harbour up to six spacers targeting a single prophage clade, while it is also possible for the same spacer to be present in up to 10 different strains (Supplementary Information S1, Table S6). The majority of unique spacers (43 out of 46) were identified to target protein-coding regions, often predicted to encode for core phage functions (terminase, portal protein, integrase) with the targeting of such regions likely to provide efficient immunity (Supplementary Information S1, Table S7).

**Figure 8:**
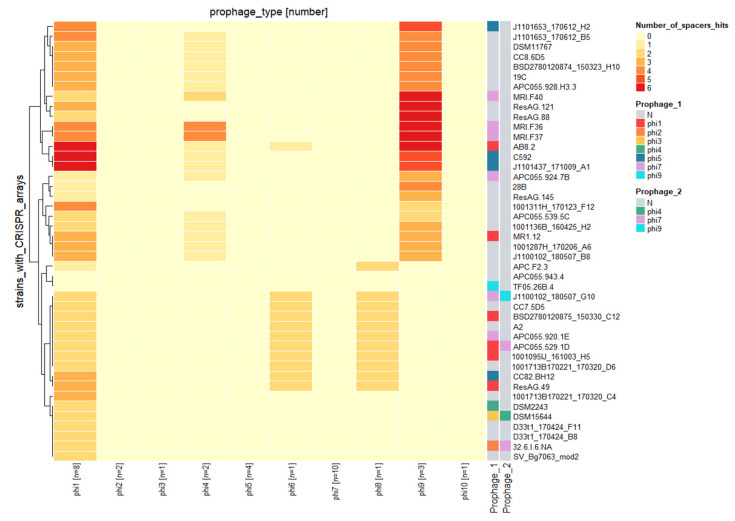
Heatmap illustrating spacer sequence hits among the CRISPR array of different *E. lenta* isolates (y-axis) and *E. lenta* prophage clades (x-axis) with the number of representatives for each clade identified in brackets. The analysis utilised 283 spacers from CRISPR arrays of 44 *E. lenta* strains identified to target prophages of the species.

The different prophage clades are not evenly targeted by the CRISPR/cas system among *E. lenta* genomes. In fact, we identified spacers of arrays targeting prophages clades 1, 4, 6, 8 and 9 while other clades seem to be unaffected. This observation is unlikely from the uneven number of prophage genomes representing each clade in this study. For example, prophage clade 1 and clade 7 are the most populated, containing 8 and 10 genomes, respectively. However, no spacers were discovered to target the latter clade (Figure 8). Likely indicating that such prophage clades, such as clade 7, possess a mechanism of resistance to CRISPR spacer targeting.

Furthermore, it is also possible for a host to harbour a spacer targeting a prophage infecting it. This was observed for isolates BSD2780120875_150330_C12, AB8#2, APC055-529-1D and MR1#12. However, only in the case of isolate AB8#2 did we identify the canonical 5′TCC PAM sequence flanking the target, suggesting that this case may represent the only example where a prophage is being actively targeted by the host CRISPR/cas system [26].

### 3.8. Evidence That Prophages Are Functional

We generated in silico and experimental evidence that indicates that the prophage elements identified in this study represent active prophage rather than domesticated elements.

**Figure 9:**
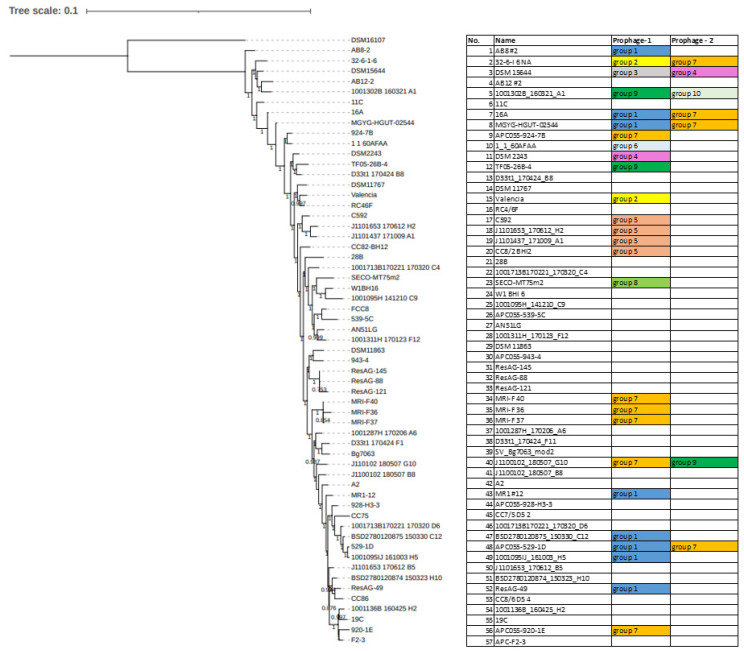
Phylogram constructed from concatenated core genes as predicted by ROARY with protein alignment using PRANK and tree constructed by FASTTree; bootstrap branch support indicated with values from 0 to 1. The table indicates the presence or absence of representatives of a particular prophage clade integrated into the genome of the bacterial isolate described.

Phylogenetic analysis of *E. lenta* strains shows that their relatedness cannot fully explain the presence of these elements among host genomes. Our phylogenetic analysis has highlighted that distantly related isolates can share prophages while those more closely related may not, showing that the gain and loss of these elements represent independent events from the evolutionary history of their hosts (Figure 9).

Analysis of read depth across prophage regions in comparison to that of the host genome show that in 16 of the 22 examined cases (isolates obtained in this study and others), prophage sequence coverage was found to be 1.1 to 5.2 times greater than that of the host (Supplementary Information S2, Figure S10). This observation was found among prophages belonging to clades 1, 2, 3, 4, and 7, implying that prophage genomes are present in higher copies than their host.

Furthermore, we could demonstrate the likely presence of virion of DSM2243phi4 when the supernatant of a culture of strain DSM2243 is subjected to genome sequencing following treatment with DNase. Sequence coverage could be seen to be 26-fold greater for the genome of DSM2243phi4 to that of the host, resulting in 44215 reads that mapped to the prophage with an average coverage of 474, compared to 442145 reads that aligned with an average coverage of 18 for the host.

The process of prophage excision results in the circularisation of its genome and restoration of the *attP* locus. We assessed the presence of the *attP* locus by PCR amplification specific for this region among prophages 14Aphi1, Valeniciaphi2, DSM2243phi4, 1-1-60FAAphi6 and 14Aphi7, representing clades 1, 2, 4, 6 and 7, respectively. This analysis verified that prophage genome circularisation could be observed in all tested cases (Supplementary Information, Figure S11), with sequenced amplicons aligning to their predicted loci for these prophages. Moreover, further confirmation of the predicted *attP* of clade 1 prophages was found with the alignment of the PCR amplicon of the *attP* to 14Aphi1 to the targeted tRNA gene (using strain DSM2243 as reference), which acts as its *attB* site of this prophage (Table 2). However, alignment of the *attP* site amplicon for those of 14Aphi7, 1-1-60FAAphi6, Valeniciaphi2 and DSM2243phi4 in a similar manner did not give any insight to their respective *attB* site.

## 4. Discussion

*E. lenta* is a bacterium of the human gastrointestinal tract implicated in the metabolism of medical and dietary compounds. To allow improved understanding of this species role in relation to human health routine strategies must be developed to enable its isolation and cultivation. To date, there is a limited description of a methodology to perform isolation of *E. lenta* from the human gut microbiome using selective growth. In this study, we devise a simple strategy utilising BHI++ medium supplemented with β-lactam antibiotics (ceftriaxone and cefotaxime). The use of this media allowed the selective isolation of *E. lenta* strains directly from human faeces (from eight of ten inspected individuals), based on their growth and characteristic colony morphology (colonies with dark yellow/brown pigmentation). Other bacteria identified to grow on this selective medium include *B. fragilis* or *B. uniformis*, both species are normally found in the gut microbiota of the human colon and have been documented to possess resistance to β-lactam antibiotics due to β-lactamase production [91,92].

To further understand the parameters that potentially influence the diversity and colonisation of *E. lenta* in the human gut, we investigated the prophages that infect this species. Genome sequencing and comparative genomics of seven newly sequenced and 50 publicly available *E. lenta* isolates allowed us to establish that 5.6% of the orthologous groups (OGs) that form the pangenome of *E. lenta* can be associated with prophages. This value is within range for prophages infecting other species of Actinobacteria, estimated at between 2% to 6.7% of OGs forming the *Bifidobacterium* pangenome [77,93].

These prophages could be placed into ten distantly related clades. Based on criteria set down by the International Committee for the Taxonomy of Viruses (ICTV) these clades achieve genus designation due to their shared nucleotide homology, gene synteny and a similar number of CDSs and tRNA genes [89]. The novelty of these prophages is striking, as indicated by their position among 12,892 phage genomes using vConTACT2. This phylogenetic analysis placed our newly identified prophages among a complex cluster of phages infecting members of the phylum Firmicutes, with their closest related genus being *Cequinduevirus* whose members infect the genus *Lactobacillus*. This analysis highlights that phage genomes that currently reside in public databases are skewed towards phages infecting bacteria that possess limited homology to those of *E. lenta*. As of 2021, NCBI virus (https://www.ncbi.nlm.nih.gov/labs/virus/vssi/#/ accessed on 2 May 2020) shows phage host entries that can be placed in the phylum Actinobacteria represent approximately 9% of total entries. However, there is only one phage entry under the class Coriobacteriia, which contains *E. lenta*. The identification of these prophage genomes should enrich public databases, enabling better identification of such phages among metagenomic studies, but also improve bioinformatic tools that allow identification of prophage sequences among bacterial genomes.

Our comparative analysis allowed the identification of a rin shufflon among one of these prophage clades (clade 1), while a DRG element was found among another five others (clades 2, 3, 4, 7 and 10). These systems appear to act on genes implicated in phage host range (likely receptor binding proteins), likely causing their diversification and influencing host range. The rin shufflon has been described among prophages of *Bifidobacterium* [76], while DGR elements have been reported to be highly prevalent in the human microbiome [94], where they are found among phages infecting species of the phyla Proteobacteria, Firmicutes and Bacteroidetes [84,95,96]. Their presence has also been indicated among phages of Actinobacteria of the human microbiome, with prophage elements of *E. lenta* having been previously flagged [96]. This study confirms their widespread prevalence among prophages infecting this species. We also obtained evidence that prophages may potentially impact host fitness, with prophages of clade 5 possessing genes implicated in exopolysaccharide biosynthesis (or possibly capsular polysaccharides).

The CRISPR/cas system of *E. lenta* has been previously demonstrated to be functional and reported to target prophages. We confirm this finding, determining that 8.3% of the total unique spacers identified among CRISPR arrays of 44 *E. lenta* genomes examined in this study target prophages of five of the ten *E. lenta* prophage clades found in this study. These prophages are not uniformly targeted by this defence system, suggesting these prophages may utilise a defence mechanism against this system. Such as the utilisation of anti CRISPR proteins documented among phages infecting species of several different bacterial families and have been found to occur among lysogenic phage of *Pseudomonas* [97,98]. These observations may explain the lack of an obvious correlation between the presence or absence of a CRISPR/cas system and the number of prophages associated with a host genome. As has been determined for other Actinobacteria genera such as *Bifidobacterium*, the presence of CRISPR/cas does not mean a bacterium genome will possess fewer prophage elements in its genome [93].

## Figures and Tables

**Table 1:** Accession numbers (DDBJ/ENA/GenBank) for genomes/plasmids of *E. lenta* isolated in this study.

No.	Isolate	Accession (Genome; Plasmids)
1	APC055-529-1D	CP089331; CP089332
2	APC055-539-5C	CP089333
3	APC055-920-1E	CP089334
4	APC055-924-7B	CP089335
5	APC055-928-H3-3	CP089336
6	APC055-943-4	CP089337
7	APC-F2-3	CP089338; CP089339, CP089340
8	DSM2243_R211	JAJQIW000000000
9	DSM2243_R611	JAJQIX000000000
10	DSM2243_R1011	JAJQKW000000000

**Table 2:** General details of identified *E. lenta* prophage genomes among host genomes.

No.	Prophage	Host (Contig Accession)	Left Boundary	Right Boundary	Size (bp)	GC%	Integration Locus (Locus Tag) Relative to Strains DSM2243 or C529	Clade
1	14Aphi1	14A (NZ_PPUR01000006.1)	29,937	71,301	41,365	67	tRNA-Leu (ELEN_RS15020)	1
2	MGYG-HGUT-02544phi1	MGYG-HGUT-02544 (NZ_CABMOO010000006.1)	29,956	71,302	41,347	67	tRNA-Leu (ELEN_RS15020)	1
3	AB8_2phi1	AB8 #2 (NZ_PPUJ01000004.1)	191,735	end of contig	>38,510	67	tRNA-Leu (ELEN_RS15020)	1
4	APC055-529-1Dphi1	APC055-529-1D (CP089331)	3,240,976	3,282,082	41,106	67	tRNA-Leu (ELEN_RS15020)	1
5	ResAG49phi1	ResAG49 (NZ_WPON01000034.1)	full contig	full contig	>30,988	67	unknown	1
6	MR1_12phi1	MR1_#12 (NZ_PPTX01000022.1)	3020	45,178	42,159	67	tRNA-Leu (ELEN_RS02880)	1
7	1001095IJ_161003_H5phi1	1001095IJ_161003_H5 (JADMUV010000007.1)	137,112	178,204	41,092	67	tRNA-Leu (ELEN_RS02880)	1
8	BSD2780120875_150330_C12phi1	BSD2780120875_150330_C12 (JADMOT010000003.1)	209,825	251,107	41,282	66	tRNA-Leu (ELEN_RS15020)	1
9	Valenciaphi2	Valencia (NZ_PPTV01000006.1)	55,915	93,457	37,543	69	tRNA-Ser (ELEN_RS00500)	2
10	32-6-I_6_NAphi2	32-6-I_6_NA (NZ_PPUM01000011.1)	26,284	63,686	37,403	69	tRNA-Ser (ELEN_RS00500)	2
11	DSM15644phi3	DSM15644 (NZ_PPUB01000019.1)	32,924	69,743	36,820	67	tRNA-Ala (ELEN_RS00055)	3
12	DSM15644phi4	DSM15644 (NZ_PPUB01000037.1)	full contig	full contig	>36,317	63	unknown	4
13	DSM2243phi4	DSM2243 (NC_013204)	3,031,719	3,068,586	36,618	63	tRNA-Ala (C592_00654)	4
14	CC82_BHI2phi5	CC82_BHI2 (NZ_PPUF01000005.1)	22,245	56,042	33,798	65	hypothetical protein (ELEN_RS14245)	5
15	C592phi5	C592 (NZ_CP021140)	500,864	534,671	33,808	65	hypothetical protein (ELEN_RS14245)	5
16	J1101437_171009_A1phi5	J1101437_171009_A1 (JADNJK010000003.1)	299,175	333,022	33,847	65	hypothetical protein (ELEN_RS14245)	5
17	J1101653_170612_H2phi5	J1101653_170612_H2 (JADPDY010000015.1)	27,299	61,125	33,826	65	hypothetical protein (ELEN_RS14245)	5
18	1-1-60AFAAphi6	1-1-60AFAA (NZ_KN214093.1)	487,345	528,047	40,703	64	unknown	6
19	APC055-529-1Dphi7	APC055-529-1D (CP089331)	3,337,687	3,371,357	33,670	59	tRNA-Arg (ELEN_RS15245)	7
20	APC055-924-7Bphi7	APC055-924-7B (CP089335)	3,608,848	3,642,543	33,695	59	tRNA-Arg (ELEN_RS15245)	7
21	14Aphi7	14A (NZ_PPUR01000011.1)	37,521	71,198	33,678	59	tRNA-Arg (ELEN_RS15245)	7
22	MGYG-HGUT-02544phi7	MGYG-HGUT-02544 (NZ_CABMOO010000011.1)	37,518	71,195	33,678	59	tRNA-Arg (ELEN_RS15245)	7
23	APC055-920-1Ephi7	APC055-920-1E (CP089334)	3,189,344	3,223,015	33,671	59	tRNA-Arg (ELEN_RS15245)	7
24	MR1-F37phi7	MRI-F37 (NZ_WPOI01000001.1)	69,802	103,502	33,701	59	tRNA-Arg (ELEN_RS15245)	7
25	MRI-F36phi7	MRI-F36 (NZ_WPOJ01000009.1)	49,797	83,505	33,709	59	tRNA-Arg (ELEN_RS15245)	7
26	32-6-1-6_NAphi7	32-6-I_6_NA (NZ_PPUM01000009.1)	46,614	46,614	33,684	59	tRNA-Arg (ELEN_RS15245)	7
27	MRI-F40phi7	MRI-F40 (NZ_WPOH01000001.1)	69,804	103,501	33,448	59	tRNA-Arg (ELEN_RS15245)	7
28	J1100102_180507_G10phi7	J1100102_180507_G10 (JADOZP010000001.1)	109,609	145,440	35,831	58	tRNA-Arg (ELEN_RS15245)	7
29	SECO-MT75m2phi8	SECO-MT75m2 (NZ_VEVP01000036.1)	3653	end of contig	>28,901	63	unknown	8
30	TF05-26B-4phi9	TF05-26B-4 (NZ_QSSL01000026.1)	16,687	end of contig	>31,589	64	unknown	9
31	J1100102_180507_G10phi9	J1100102_180507_G10 (JADOZP010000013.1)	10,288	46,514	36,226	65	unknown	9
32	1001302B_160321_A1phi9	1001302B_160321_A1 (JADNIO010000007.1)	165,588	202,064	36,476	65	unknown	9
33	1001302B_160321_A1phi10	1001302B_160321_A1 (JADNIO010000005.1)	220,405	252,147	31,742	58	unknown	10

**Table 3:** Putative insertion sites among clade 1 *E. lenta* prophages.

Prophage	Predicted attP-Site	attB Relative to DSM2243 (Locus)
14Aphi1	CAACCCCATGGAGGTTCAAGTCCTCTCGCCCGCACCATCTGAA	tRNA-Leu (ELEN_RS15020)
MGYG-HGUT-02544phi1	AACCCCATGGAGGTTCAAGTCCTCTCGCCCGCACCATCTGAA	tRNA-Leu (ELEN_RS15020)
APC055-529-1Dphi1	TTCAGATGGTGCGGGCGAGAGGACTTGAACCTCCATGGGGTT	tRNA-Leu (ELEN_RS15020)
1001095IJ_161003_H5phi1	ACTTAAAATCTTCCGGCTTCGGCCTTGCGGGTTCGAGTCCCGCCGCCCCTACCA	tRNA-Leu (ELEN_RS02880)
BSD2780120875_150330_C12phi1	TTCAGATGGTGCGGGCGAGAGGACTTGAACCTCCATGGGGTT	tRNA-Leu (ELEN_RS15020)

## Data Availability

Not applicable.

## Supplementary Materials

The following are available online at https://www.mdpi.com/article/10.3390/microorganisms10010195/s1. Figure S1. Colony morphologies observed for presumptive *E. lenta* colonies. Figure S2. *E. lenta* isolates APC-FCC8 and APC055-920-1E Gram stained and examined under light microscopy. Figure S3. ANI % of 57 *E. lenta* genomes and *E. sinesis* DSM16107. Figure S4. Presence/absence heatmap of genes among *E. lenta* isolates involved in the activation/inactivation of drugs and dietary compounds. Figure S5. VICTOR-generated phylogenomic Genome-BLAST Distance Phylogeny (GBDP) trees of *E. lenta* prophages. Figure S6. Number OGs annotated with a particular function found in pangenome of *E. lenta* prophages. Figure S7. A maximum likelihood phylogenetic of the large terminase proteins of prophage belonging to different prophage clades of *E. lenta*. Figure S8. Analysis of *E. lenta* prophages using VIPtree, which indicates a distant relationship with phages of the genus Cequinduevirus. Figure S9. Comparison of the genomes of *L. lenta* clade 9 prophages with Lactobacillus phage c5 employing TBLASTX and visualised with Easyfig. Figure S10. Ratio of median coverage of different prophage vs median coverage of their entire bacterial host’s genome. Figure S11. PCR targeting at attP of prophages among different strains of *E. lenta*. Table S1: Details of strains utilised in this study for experimental and genomic analysis. Table S2. Details for *E. lenta* isolates obtained in this study. Table S3: Primers utilised for PCRs conducted in this study. Table S4. Determined MIC values of cefotaxime and ceftriaxone against *E. lenta*. Table S5. Morphology prediction of *E. lenta* prophages based on VirFam. Table S4. Viral clusters of *E. lenta* prophages as defined by vconTACT2 analysis. Table S7. Presence of spacers among 42 *E. lenta* isolates target prophages and regions they target.

## References

[1] Shkoporov A.N., Hill C. (2019). Bacteriophages of the Human Gut: The “Known Unknown” of the Microbiome. Cell Host Microbe.

[2] Sender R., Fuchs S., Milo R. (2016). Revised Estimates for the Number of Human and Bacteria Cells in the Body. PLoS Biol..

[3] Rowland I., Gibson G., Heinken A., Scott K., Swann J., Thiele I., Tuohy K. (2018). Gut microbiota functions: Metabolism of nutrients and other food components. Eur. J. Nutr..

[4] Koppel N., Rekdal V.M., Balskus E.P. (2017). Chemical transformation of xenobiotics by the human gut microbiota. Science.

[5] Spanogiannopoulos P., Bess E.N., Carmody R.N., Turnbaugh P.J. (2016). The microbial pharmacists within us: A metagenomic view of xenobiotic metabolism. Nat. Rev. Microbiol..

[6] Haiser H.J., Turnbaugh P.J. (2013). Developing a metagenomic view of xenobiotic metabolism. Pharmacol. Res..

[7] Wade W.G., Downes J., Dymock D., Hiom S.J., Weightman A.J., Dewhirst F.E., Paster B.J., Tzellas N., Coleman B. (1999). The family Coriobacteriaceae: Reclassification of Eubacterium exiguum (Poco et al, 1996) and Peptostreptococcus heliotrinreducens (Lanigan 1976) as Slackia exigua gen. nov., comb. nov. and Slackia heliotrinireducens gen. nov., comb. nov., and Eubacterium. Int. J. Syst. Bacteriol..

[8] Gupta R.S., Chen W.J., Adeolu M., Chai Y. (2013). Molecular signatures for the class Coriobacteriia and its different clades; proposal for division of the class Coriobacteriia into the emended order Coriobacteriales, containing the emended family Coriobacteriaceae and Atopobiaceae fam. nov., and Eggerthe. Int. J. Syst. Evol. Microbiol..

[9] Sousa T., Paterson R., Moore V., Carlsson A., Abrahamsson B., Basit A.W. (2008). The gastrointestinal microbiota as a site for the biotransformation of drugs. Int. J. Pharm..

[10] Kageyama A., Benno Y., Nakase T. (1999). Phylogenetic evidence for the transfer of *Eubacterium lentum* to the genus Eggerthella as *Eggerthella lenta* gen. nov., comb. nov. Int. J. Syst. Bacteriol..

[11] Koppel N., Bisanz J.E., Pandelia M.E., Turnbaugh P.J., Balskus E.P. (2018). Discovery and characterization of a prevalent human gut bacterial enzyme sufficient for the inactivation of a family of plant toxins. Elife.

[12] Haiser H.J., Gootenberg D.B., Chatman K., Sirasani G., Balskus E.P., Turnbaugh P.J. (2013). Predicting and manipulating cardiac drug inactivation by the human gut bacterium *Eggerthella lenta* + NIH Public Access. Science.

[13] Lindenbaum J., Rund D.G., Butler V.P., Tse-Eng D., Saha J.R. (1981). Inactivation of Digoxin by the Gut Flora: Reversal by Antibiotic Therapy. N. Engl. J. Med..

[14] Rekdal V.M., Bess E.N., Bisanz J.E., Turnbaugh P.J., Balskus E.P. (2019). Discovery and inhibition of an interspecies gut bacterial pathway for Levodopa metabolism. Science.

[15] Jung C.M., Heinze T.M., Schnackenberg L.K., Mullis L.B., Elkins S.A., Elkins C.A., Steele R.S., Sutherland J.B. (2009). Interaction of dietary resveratrol with animal-associated bacteria. FEMS Microbiol. Lett..

[16] Bess E.N., Bisanz J.E., Yarza F., Bustion A., Rich B.E., Li X., Kitamura S., Waligurski E., Ang Q.Y., Alba D.L. (2020). Genetic basis for the cooperative bioactivation of plant lignans by *Eggerthella lenta* and other human gut bacteria. Nat. Microbiol..

[17] Elias R.M., Khoo S.Y., Pupaibool J., Nienaber J.H., Cummins N.W. (2012). Multiple pyogenic liver abscesses caused by *Eggerthella lenta* treated with ertapenem: A case report. Case Rep. Med..

[18] Gardiner B.J., Tai A.Y., Kotsanas D., Francis M.J., Roberts S.A., Ballard S.A., Junckerstorff R.K., Kormana T.M. (2015). Clinical and microbiological characteristics of *Eggerthella lenta* bacteremia. J. Clin. Microbiol..

[19] Venugopal A.A., Szpunar S., Johnson L.B. (2012). Risk and prognostic factors among patients with bacteremia due to *Eggerthella lenta*. Anaerobe.

[20] Hsu B.B., Gibson T.E., Yeliseyev V., Liu Q., Lyon L., Bry L., Silver P.A., Gerber G.K. (2019). Dynamic Modulation of the Gut Microbiota and Metabolome by Bacteriophages in a Mouse Model. Cell Host Microbe.

[21] Manrique P., Dills M., Young M.J. (2017). The Human Gut Phage Community and Its Implications for Health and Disease. Viruses.

[22] Howard-Varona C., Hargreaves K.R., Abedon S.T., Sullivan M.B. (2017). Lysogeny in nature: Mechanisms, impact and ecology of temperate phages. ISME J..

[23] Cumby N., Davidson A.R., Maxwell K.L. (2012). The moron comes of age. Bacteriophage.

[24] Bondy-Denomy J., Davidson A.R. (2014). When a Virus is not a Parasite: The Beneficial Effects of Prophages on Bacterial Fitness. J. Microbiol..

[25] Canchaya C., Fournous G., Chibani-Chennoufi S., Dillmann M.L., Brüssow H. (2003). Phage as agents of lateral gene transfer. Curr. Opin. Microbiol..

[26] Soto-Perez P., Bisanz J.E., Berry J.D., Lam K.N., Bondy-Denomy J., Turnbaugh P.J. (2019). CRISPR-Cas System of a Prevalent Human Gut Bacterium Reveals Hyper-targeting against Phages in a Human Virome Catalog. Cell Host Microbe.

[27] Bisanz J.E., Soto-Perez P., Lam K.N., Bess E.N., Haiser H.J., Allen-Vercoe E., Rekdal V.M., Balskus E.P., Turnbaugh P.J. (2018). Illuminating the microbiome’s dark matter: A functional genomic toolkit for the study of human gut Actinobacteria. BioRxiv.

[28] Bolger A.M.M., Lohse M., Usadel B. (2014). Genome analysis Trimmomatic: A flexible trimmer for Illumina sequence data. Bioinformatics.

[29] Gurevich A., Saveliev V., Vyahhi N., Tesler G. (2013). QUAST: Quality assessment tool for genome assemblies. Bioinformatics.

[30] Bankevich A., Nurk S., Antipov D., Gurevich A.A.A., Dvorkin M., Kulikov A.S.S., Lesin V.M.M., Nikolenko S.I.I., Pham S., Prjibelski A.D.D. (2012). SPAdes: A New Genome Assembly Algorithm and Its Applications to Single-Cell Sequencing. J. Comput. Biol..

[31] Parks D.H., Imelfort M., Skennerton C.T., Hugenholtz P., Tyson G.W. (2015). CheckM: Assessing the quality of microbial genomes recovered from isolates, single cells, and metagenomes. Genome Res..

[32] Wick R.R., Judd L.M., Gorrie C.L., Holt K.E. (2017). Unicycler: Resolving bacterial genome assemblies from short and long sequencing reads. PLoS Comput. Biol..

[33] Li H. (2018). Minimap2: Pairwise alignment for nucleotide sequences. Bioinformatics.

[34] Langmead B., Salzberg S.L. (2012). Fast gapped-read alignment with Bowtie 2. Nat. Methods.

[35] Li H., Handsaker B., Wysoker A., Fennell T., Ruan J., Homer N., Marth G., Abecasis G., Durbin R. (2009). The Sequence Alignment/Map format and SAMtools. Bioinformatics.

[36] Marçais G., Delcher A.L., Phillippy A.M., Coston R., Salzberg S.L., Zimin A. (2018). MUMmer4: A fast and versatile genome alignment system. PLoS Comput. Biol..

[37] Pritchard L., Glover R.H., Humphris S., Elphinstone J.G., Toth I.K. (2016). Genomics and taxonomy in diagnostics for food security: Soft-rotting enterobacterial plant pathogens. Anal. Methods.

[38] Seemann T. (2014). Prokka: Rapid prokaryotic genome annotation. Bioinformatics.

[39] Lechner M., Findeiß S., Steiner L., Marz M., Stadler P.F.P.F., Prohaska S.J.S.J. (2011). Proteinortho: Detection of (Co-)orthologs in large-scale analysis. BMC Bioinform..

[40] Zhao Y., Jia X., Yang J., Ling Y., Zhang Z., Yu J., Wu J., Xiao J. (2014). PanGP: A tool for quickly analyzing bacterial pan-genome profile. Bioinformatics.

[41] Tettelin H., Riley D., Cattuto C., Medini D. (2008). Comparative genomics: The bacterial pan-genome. Curr. Opin. Microbiol..

[42] Galperin M.Y., Wolf Y.I., Makarova K.S., Vera Alvarez R., Landsman D., Koonin E.V. (2021). COG database update: Focus on microbial diversity, model organisms, and widespread pathogens. Nucleic Acids Res..

[43] Wu S., Zhu Z., Fu L., Niu B., Li W. (2011). WebMGA: A customizable web server for fast metagenomic sequence analysis. BMC Genom..

[44] Löytynoja A. (2014). Phylogeny-aware alignment with PRANK. Methods Mol. Biol..

[45] Page A.J., Cummins C.A., Hunt M., Wong V.K., Reuter S., Holden M.T.G., Fookes M., Falush D., Keane J.A., Parkhill J. (2015). Roary: Rapid large-scale prokaryote pan genome analysis. Bioinformatics.

[46] Price M.N., Dehal P.S., Arkin A.P. (2010). FastTree 2-Approximately maximum-likelihood trees for large alignments. PLoS ONE.

[47] Darling A.E., Mau B., Perna N.T. (2010). progressiveMauve: Multiple genome alignment with gene gain, loss and rearrangement. PLoS ONE.

[48] Ågren J., Sundström A., Håfström T., Segerman B. (2012). Gegenees: Fragmented alignment of multiple genomes for determining phylogenomic distances and genetic signatures unique for specified target groups. PLoS ONE.

[49] Meier-Kolthoff J.P., Göker M. (2017). VICTOR: Genome-based phylogeny and classification of prokaryotic viruses. Bioinformatics.

[50] Kumar S., Stecher G., Li M., Knyaz C., Tamura K. (2018). MEGA X: Molecular evolutionary genetics analysis across computing platforms. Mol. Biol. Evol..

[51] Edgar R.C. (2004). MUSCLE: Multiple sequence alignment with high accuracy and high throughput. Nucleic Acids Res..

[52] Jones D.T., Taylor W.R., Thornton J.M. (1992). The rapid generation of mutation data matrices from protein sequences. Bioinformatics.

[53] Letunic I., Bork P. (2019). Interactive Tree Of Life (iTOL) v4: Recent updates and new developments. Nucleic Acids Res..

[54] Bin Jang H., Bolduc B., Zablocki O., Kuhn J.H., Roux S., Adriaenssens E.M., Brister J.R., Kropinski A.M., Krupovic M., Lavigne R. (2019). Taxonomic assignment of uncultivated prokaryotic virus genomes is enabled by gene-sharing networks. Nat. Biotechnol..

[55] Otasek D., Morris J.H., Bouças J., Pico A.R., Demchak B. (2019). Cytoscape Automation: Empowering workflow-based network analysis. Genome Biol..

[56] Hyatt D., Chen G.L., LoCascio P.F., Land M.L., Larimer F.W., Hauser L.J. (2010). Prodigal: Prokaryotic gene recognition and translation initiation site identification. BMC Bioinform..

[57] Nishimura Y., Yoshida T., Kuronishi M., Uehara H., Ogata H., Goto S. (2017). ViPTree: The viral proteomic tree server. Bioinformatics.

[58] Arndt D., Grant J.R.J.R., Marcu A., Sajed T., Pon A., Liang Y., Wishart D.S.D.S. (2016). PHASTER: A better, faster version of the PHAST phage search tool. Nucleic Acids Res..

[59] Darling A.C.E., Mau B., Blattner F.R., Perna N.T. (2004). Mauve: Multiple alignment of conserved genomic sequence with rearrangements. Genome Res..

[60] Eddy S.R. (2011). Accelerated Profile HMM Searches. PLoS Comput. Biol..

[61] Grazziotin A.L., Koonin E.V., Kristensen D.M. (2017). Prokaryotic virus orthologous groups (pVOGs): A resource for comparative genomics and protein family annotation. Nucleic Acids Res..

[62] Jones P., Binns D., Chang H.-Y.H.Y.Y., Fraser M., Li W., McAnulla C., McWilliam H., Maslen J., Mitchell A., Nuka G. (2014). InterProScan 5: Genome-scale protein function classification. Bioinformatics.

[63] Lopes A., Tavares P., Petit M.A., Guérois R., Zinn-Justin S. (2014). Automated classification of tailed bacteriophages according to their neck organization. BMC Genom..

[64] Söding J., Biegert A., Lupas A.N., Soding J., Biegert A., Lupas A.N. (2005). The HHpred interactive server for protein homology detection and structure prediction. Nucleic Acids Res..

[65] Camacho C., Coulouris G., Avagyan V., Ma N., Papadopoulos J., Bealer K., Madden T.L. (2009). BLAST+: Architecture and applications. BMC Bioinform..

[66] Clavel T., Lepage P., Charrier C. (2014). The family Coriobacteriaceae. The Prokaryotes: Actinobacteria.

[67] Danylec N., Stoll D.A., Göbl A., Huch M. (2020). Draft Genome Sequences of 13 Isolates of *Adlercreutzia equolifaciens*, *Eggerthella lenta*, and *Gordonibacter urolithinfaciens*, Isolated from Human Fecal Samples in Karlsruhe, Germany. Microbiol. Resour. Announc..

[68] Dobkin J.F., Saha, J.R., Butler V.P., Neu H.C., Lindenbaum J. (1983). Digoxin-inactivating bacteria: Identification in human gut flora. Science.

[69] Clavel T., Henderson G., Alpert C.A., Philippe C., Rigottier-Gois L., Doré J., Blaut M. (2005). Intestinal bacterial communities that produce active estrogen-like compounds enterodiol and enterolactone in humans. Appl. Environ. Microbiol..

[70] Liderot K., Larsson M., Boräng S., Özenci V. (2010). Polymicrobial bloodstream infection with *Eggerthella lenta* and *Desulfovibrio desulfuricans*. J. Clin. Microbiol..

[71] Bisanz J.E., Soto-Perez P., Noecker C., Aksenov A.A., Lam K.N., Kenney G.E., Bess E.N., Haiser H.J., Kyaw T.S., Yu F.B. (2020). A Genomic Toolkit for the Mechanistic Dissection of Intractable Human Gut Bacteria. Cell Host Microbe.

[72] Soto Perez P.A. (2021). Plasmids, Immunity, and Phages of Gut Bacterium Eggerthella Lenta.

[73] Rasko D.A., Rosovitz M.J., Myers G.S.A., Mongodin E.F., Fricke W.F., Gajer P., Crabtree J., Sebaihia M., Thomson N.R., Chaudhuri R. (2008). The pangenome structure of Escherichia coli: Comparative genomic analysis of E. coli commensal and pathogenic isolates. J. Bacteriol..

[74] Arboleya S., Bottacini F., O’Connell-Motherway M., Ryan C.A., Ross R.P., van Sinderen D., Stanton C. (2018). Gene-trait matching across the Bifidobacterium longum pan-genome reveals considerable diversity in carbohydrate catabolism among human infant strains. BMC Genom..

[75] Declerck B., Van der Beken Y., De Geyter D., Piérard D., Wybo I. (2021). Antimicrobial susceptibility testing of *Eggerthella lenta* blood culture isolates at a university hospital in Belgium from 2004 to 2018. Anaerobe.

[76] Mavrich T.N., Casey E., Oliveira J., Bottacini F., James K., Franz C.M.A.P., Lugli G.A., Neve H., Ventura M., Hatfull G.F. (2018). Characterization and induction of prophages in human gut-associated Bifidobacterium hosts. Sci. Rep..

[77] Lugli G.A., Milani C., Turroni F., Tremblay D., Ferrario C., Mancabelli L., Duranti S., Ward D.V., Ossiprandi M.C., Moineau S. (2016). Prophages of the genus Bifidobacterium as modulating agents of the infant gut microbiota. Environ. Microbiol..

[78] Pope W.H., Jacobs-Sera D., Russell D.A., Peebles C.L., Al-Atrache Z., Alcoser T.A., Alexander L.M., Alfano M.B., Alford S.T., Amy N.E. (2011). Expanding the Diversity of Mycobacteriophages: Insights into Genome Architecture and Evolution. PLoS ONE.

[79] Oliveira L., Tavares P., Alonso J.C. (2013). Headful DNA packaging: Bacteriophage SPP1 as a model system. Virus Res..

[80] Ellis D.M., Dean D.H. (1985). Nucleotide sequence of the cohesive single-stranded ends of Bacillus subtilis temperate bacteriophage phi 105. J. Virol..

[81] Lillehaug D., Lindqvist B.H., Birkeland N.K. (1991). Characterization of φLC3, a Lactococcus lactis subsp. cremoris temperate bacteriophage with cohesive single-stranded DNA ends. Appl. Environ. Microbiol..

[82] Barr J.J., Auro R., Furlan M., Whiteson K.L., Erb M.L., Pogliano J., Stotland A., Wolkowicz R., Cutting A.S., Doran K.S. (2013). Bacteriophage adhering to mucus provide a non-host-derived immunity. Proc. Natl. Acad. Sci. USA.

[83] Cho G.-S., Ritzmann F., Eckstein M., Huch M., Briviba K., Behsnilian D., Neve H., Franz C.M.A.P. (2016). Quantification of Slackia and *Eggerthella* spp. in Human Feces and Adhesion of Representatives Strains to Caco-2 Cells. Front. Microbiol..

[84] Liu M., Deora R., Doulatov S.R., Gingery M., Eiserling F.A., Preston A., Maskell D.J., Simons R.W., Cotter P.A., Parkhill J. (2016). Reverse Transcriptase-Mediated Tropism Switching in Bordetella Bacteriophage. Science.

[85] Alayyoubi M., Guo H., Dey S., Golnazarian T., Brooks G.A., Rong A., Miller J.F., Ghosh P. (2013). Structure of the essential diversity-generating retroelement protein bAvd and its functionally important interaction with reverse transcriptase. Structure.

[86] Guo H., Arambula D., Ghosh P., Miller J.F. (2015). Diversity-generating Retroelements in Phage and Bacterial Genomes. Mobile DNA III.

[87] Dai W., Hodes A., Hui W.H., Gingery M., Miller J.F., Zhou Z.H. (2010). Three-dimensional structure of tropism-switching Bordetella bacteriophage. Proc. Natl. Acad. Sci. USA.

[88] Dziewit L., Jazurek M., Drewniak L., Baj J., Bartosik D. (2007). The SXT conjugative element and linear prophage N15 encode toxin-antitoxin-stabilizing systems homologous to the tad-ata module of the Paracoccus aminophilus plasmid pAMI2. J. Bacteriol..

[89] Adriaenssens E.M., Rodney Brister J. (2017). How to name and classify your phage: An informal guide. Viruses.

[90] Riipinen K.A., Forsman P., Alatossava T. (2011). The genomes and comparative genomics of Lactobacillus delbrueckii phages. Arch. Virol..

[91] Edwards R. (1997). Resistance to β-lactam antibiotics in bacteroides spp.. J. Med. Microbiol..

[92] Hedberg M., Lindqvist L., Bergman T., Nord C.E. (1995). Purification and characterization of a new β-lactamase from Bacteroides uniformis. Antimicrob. Agents Chemother..

[93] Wang G., Liu Q., Pei Z., Wang L., Tian P., Liu Z., Zhao J., Zhang H., Chen W. (2020). The Diversity of the CRISPR-Cas System and Prophages Present in the Genome Reveals the Co-evolution of Bifidobacterium pseudocatenulatum and Phages. Front. Microbiol..

[94] Yan F., Yu X., Duan Z., Lu J., Jia B., Qiao Y., Sun C., Wei C. (2019). Discovery and characterization of the evolution, variation and functions of diversity-generating retroelements using thousands of genomes and metagenomes. BMC Genom..

[95] Cornuault J.K., Petit M.-A., Mariadassou M., Benevides L., Moncaut E., Langella P., Sokol H., De Paepe M. (2018). Phages infecting Faecalibacterium prausnitzii belong to novel viral genera that help to decipher intestinal viromes. Microbiome.

[96] Benler S., Cobián-Güemes A.G., McNair K., Hung S.H., Levi K., Edwards R., Rohwer F. (2018). A diversity-generating retroelement encoded by a globally ubiquitous Bacteroides phage 06 Biological Sciences 0605 Microbiology. Microbiome.

[97] Bondy-Denomy J., Pawluk A., Maxwell K.L., Davidson A.R. (2013). Bacteriophage genes that inactivate the CRISPR/Cas bacterial immune system. Nature.

[98] Stanley S.Y., Maxwell K.L. (2018). Phage-Encoded Anti-CRISPR Defenses. Annu. Rev. Genet..

