# Supplementary material for: Selective Isolation of Eggerthella lenta from Human Faeces and Characterisation of the Species Prophage Diversity
Source: Microorganisms. 2022 Jan 17;10(1):195. doi: 10.3390/microorganisms10010195 (PMC8778435; doi:10.3390/microorganisms10010195)
Supplement: Supplementary file 1 [file microorganisms-10-00195-s001.zip › Supplementary Information S2.pdf]

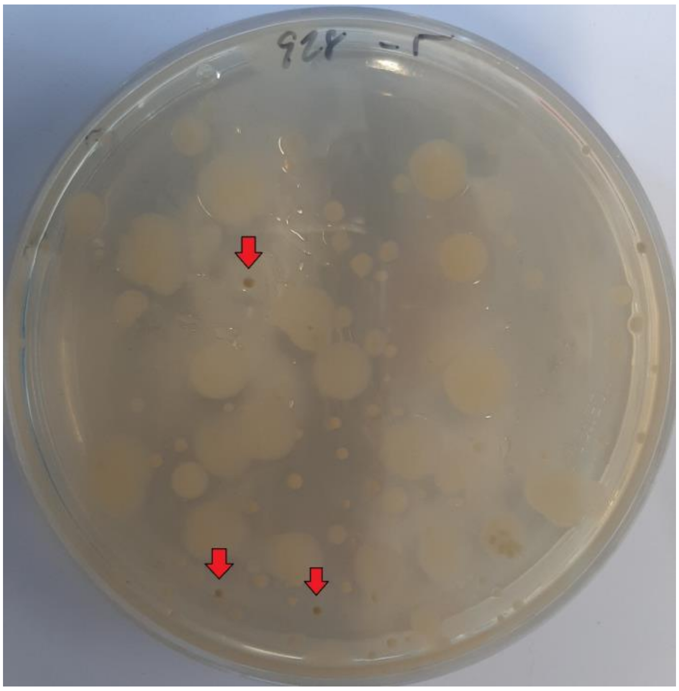

Figure S1. Colony morphologies observed for presumptive *E. lenta* colonies (red arrows) when grown on BHI++ agar (with overlay) supplemented with ceftriaxone and cefotaxime.

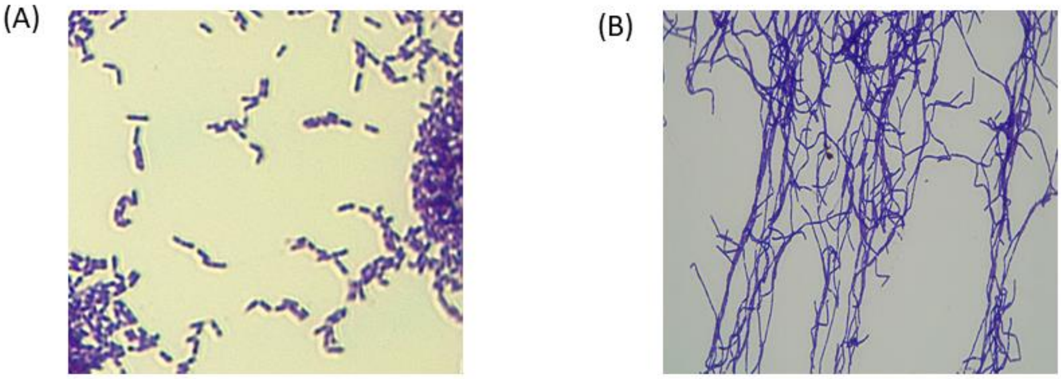

Figure S2. *E. lenta* isolates (**A**) APC-FCC8 and (**B**) APC055-920-1E Gram stained and examined under light microscopy (magnification X1000)

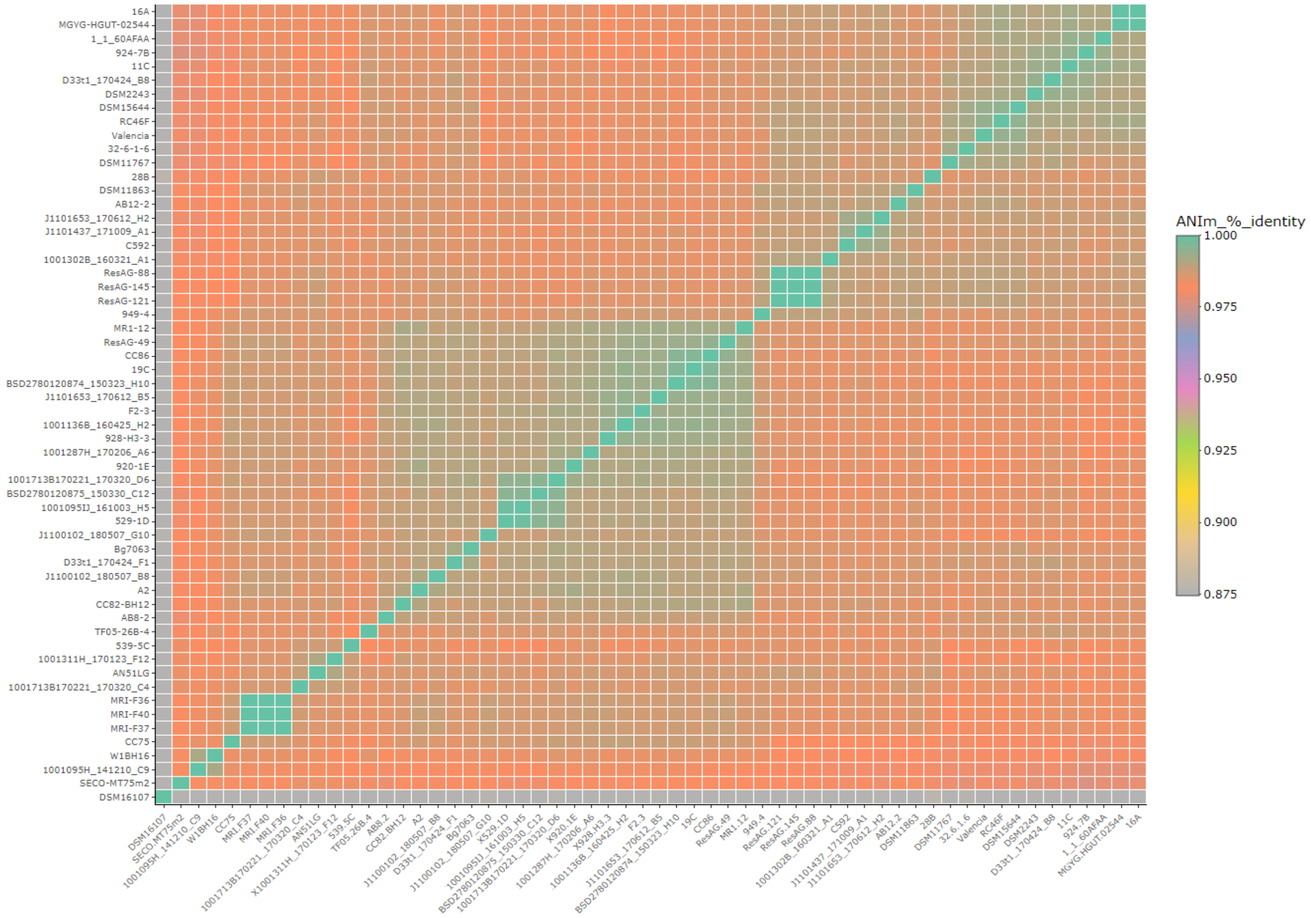

Figure S3. ANI % of 57 *E. lenta* genomes and *E. sinesis* DSM16107

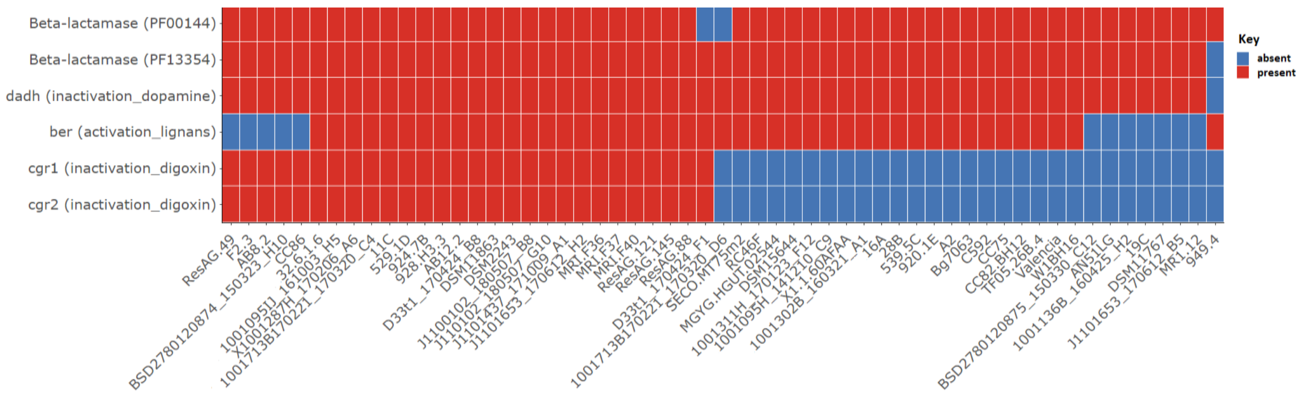

Figure S4. Presence/absence heatmap of genes among *E. lenta* isolates involved in the activation/inactivation of drugs and dietary compounds as well as genes implicated in β-lactam resistance

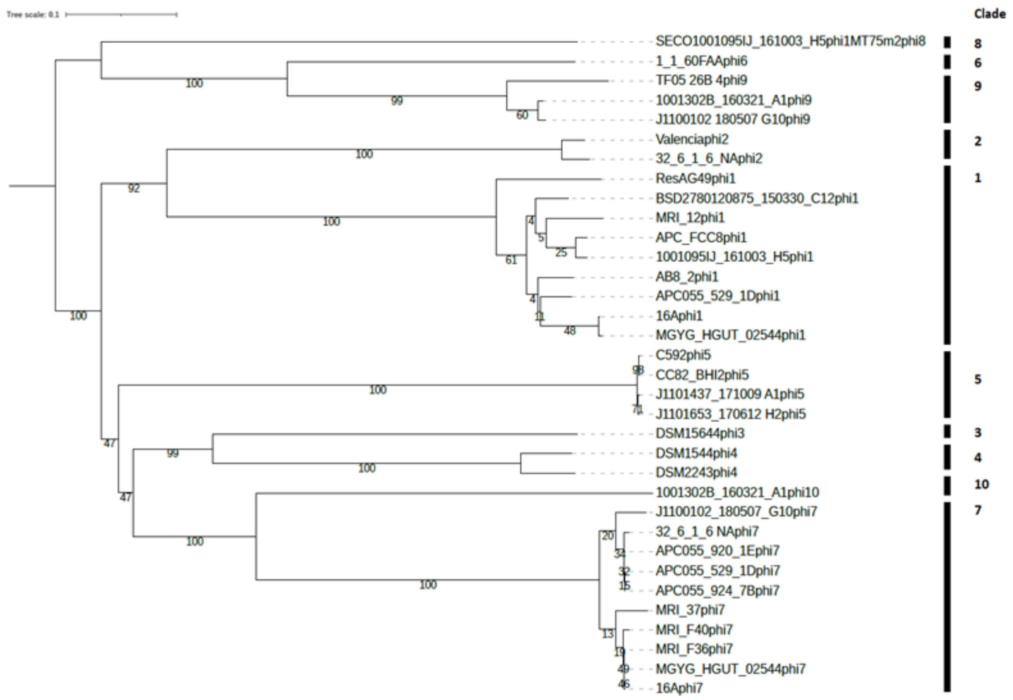

Figure S5. VICTOR-generated phylogenomic Genome-BLAST Distance Phylogeny (GBDP) trees of *E. lenta* prophages of using the formula D6. The numbers above branches are GBDP pseudo-bootstrap support values from 100 replications. The 10 different clades of prophage identified is illustrated.

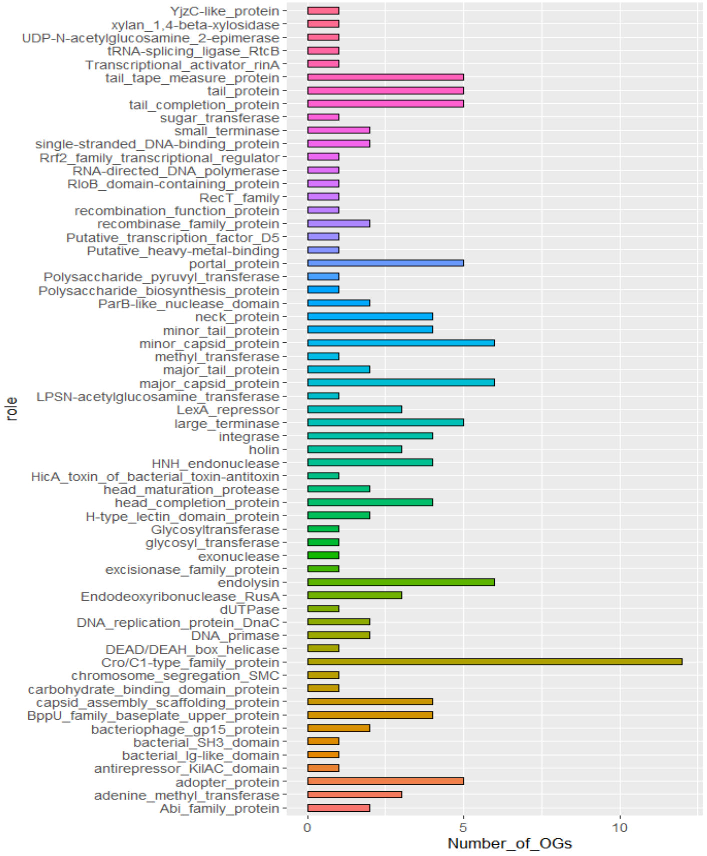

Figure S6. Number OGs annotated with a particular function found in pangenome of *E. lenta* prophages.

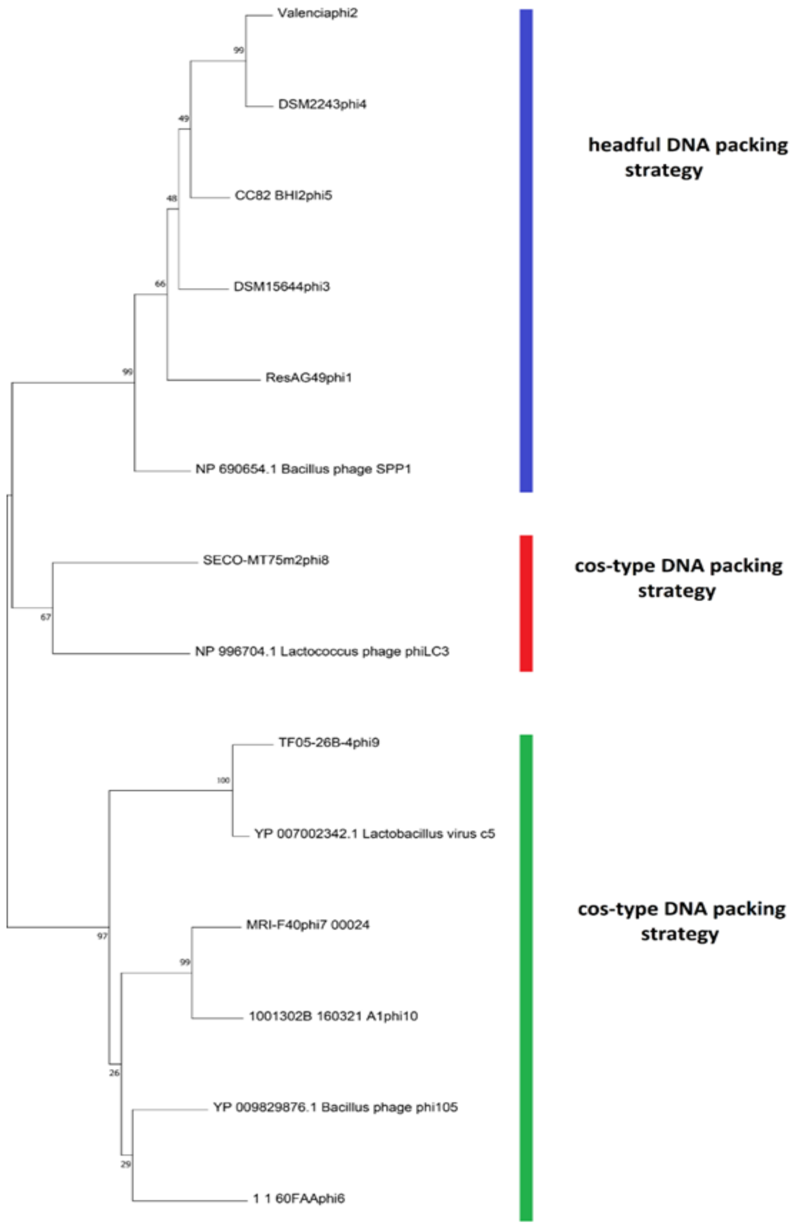

Figure S7. A maximum likelihood phylogenetic tree of the large terminase proteins of prophage belonging to different prophage clades infecting *E. lenta* compared to those of *Bacillus* phage SPP1, *Lactococcus* phage phiLC3, *Lactobacillus* phage phage c5 and *Bacillus* phage phi105, with 1000 bootstrap replicates. Predicted DNA packing strategy of the large terminases of phages and prophages are illustrated.

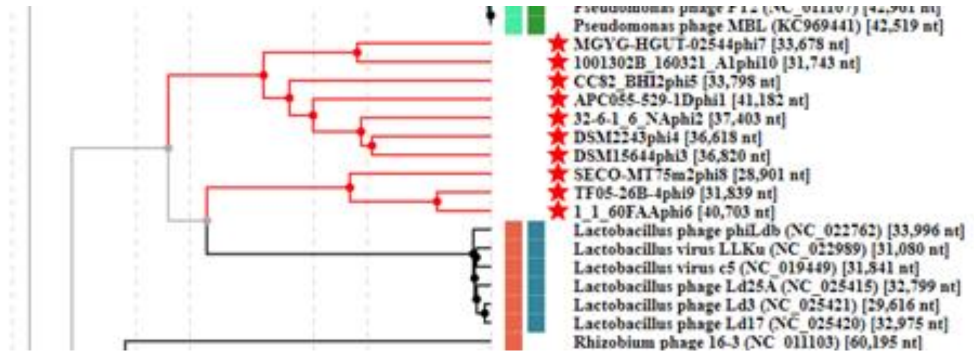

Figure S8. Analysis of *E. lenta* prophages using VIPtree (v2) which indicates distant relationship with phages of the genus *Cequinduevirus*.

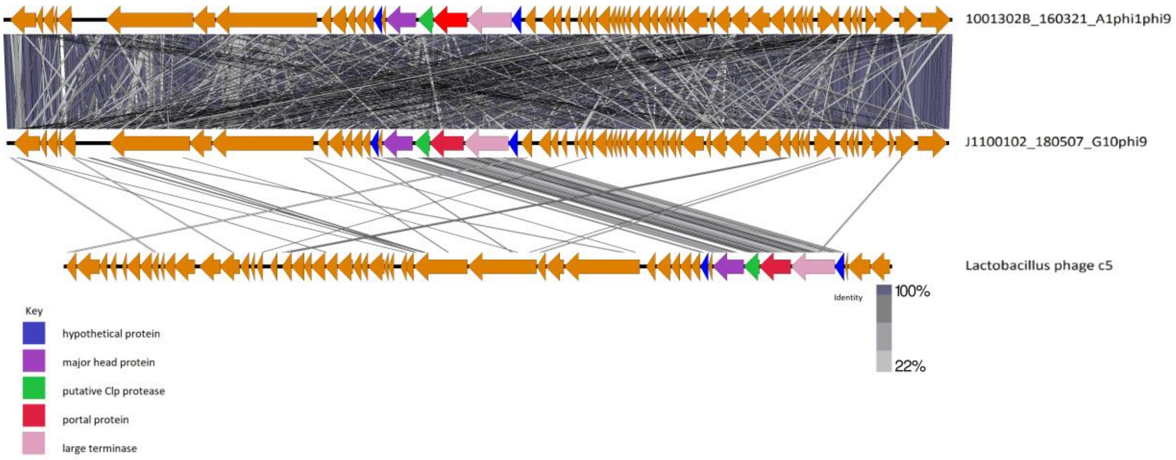

Figure S9. Comparison of the genomes of *E. lenta* group 9 prophages with *Lactobacillus* phage c5 employing TBLASTX and visualized with Easyfig. Genome maps comprise of arrows indicating the locations of open reading frames (ORFs) among the different phage genomes. Arrows have been color-coded describing their predicted roles (see key), and lines between genome maps indicate the level of Identity.

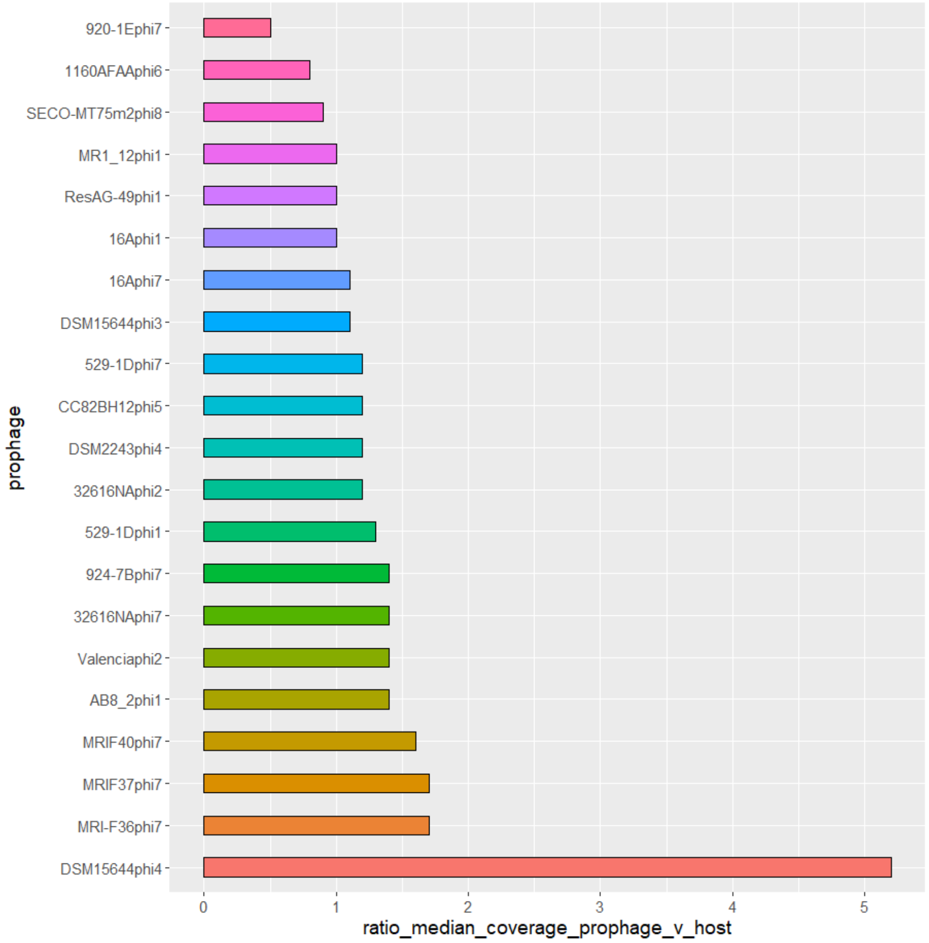

Figure S10. Ratio of median coverage of different prophage vs median coverage of their entire bacterial host's genome.

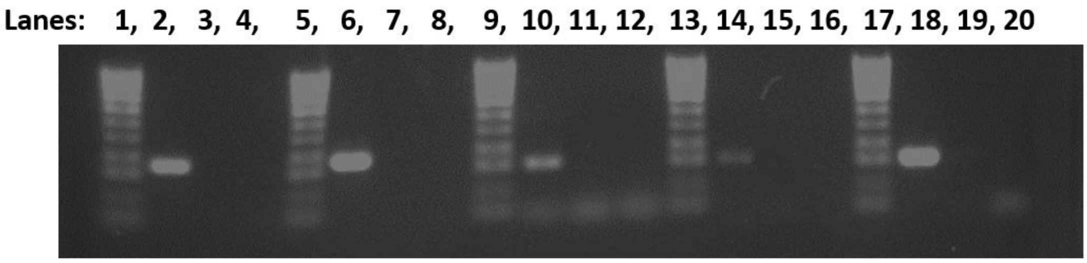

Figure S11. PCR targeting at attP of prophages among different strains of *E. lenta*, which confirms prophage genomes can excise from that of host and circularise. Testing for presence of attP site for prophages 14Aphi1, 14Aphi7, 1-1-60FAAphi6, Valeniciaphi2 and DSM2243phi4 on host strain; Lanes 2, 6, 10, 14 & 18. Negative control, testing of attP site of prophages in non-host strain (DSM2243 or 14A); Lanes 3, 7, 11, 15 & 19. PCR control (water); Lanes 4, 8, 12, 16 & 20. DNA marker (Hyperladder 100 bp, Bioline); Lanes 1, 5, 9, 13 & 17.
